# Photosensitive ion channels in layered MXene membranes modified with plasmonic gold nanostars and cellulose nanofibers

**DOI:** 10.1038/s41467-023-36039-5

**Published:** 2023-01-23

**Authors:** Jeonghee Yeom, Ayoung Choe, Jiyun Lee, Jeeyoon Kim, Jinyoung Kim, Seung Hak Oh, Cheolhong Park, Sangyun Na, Young-Eun Shin, Youngoh Lee, Yun Goo Ro, Sang Kyu Kwak, Hyunhyub Ko

**Affiliations:** 1 School of Energy and Chemical Engineering, Ulsan National Institute of Science and Technology (UNIST), Ulsan Metropolitan City, 44919 Republic of Korea; 2 Department of Chemical and Biological Engineering, Korea University, 145 Anam-ro, Seongbuk-gu, Seoul 02841 Republic of Korea

**Keywords:** Two-dimensional materials, Two-dimensional materials, Materials for devices

## Abstract

Ion channels transduce external stimuli into ion-transport-mediated signaling, which has received considerable attention in diverse fields such as sensors, energy harvesting devices, and desalination membrane. In this work, we present a photosensitive ion channel based on plasmonic gold nanostars (AuNSs) and cellulose nanofibers (CNFs) embedded in layered MXene nanosheets. The MXene/AuNS/CNF (MAC) membrane provides subnanometer-sized ionic pathways for light-sensitive cationic flow. When the MAC nanochannel is exposed to NIR light, a photothermal gradient is formed, which induces directional photothermo-osmotic flow of nanoconfined electrolyte against the thermal gradient and produces a net ionic current. MAC membrane exhibits enhanced photothermal current compared with pristine MXene, which is attributed to the combined photothermal effects of plasmonic AuNSs and MXene and the widened interspacing of the MAC composite via the hydrophilic nanofibrils. The MAC composite membranes are envisioned to be applied in flexible ionic channels with ionogels and light-controlled ionic circuits.

## Abstract (web summary)

Artificial ion channels are in demand for ionotronic devices. Here, the authors use layered MXene membranes modified with plasmonic gold nanostars and cellulose nanofibers to convert a thermal gradient into an ion current for photosensitive ion channeling.

## Introduction

Ions, the essential sources of signaling tools for living organisms, have recently attracted considerable attention as current carriers that substitute electrons to address the signal mismatch between electronic devices and ionic biological features. Ionotronics have applications in the human–machine interfaces of epidermal and implantable electronic devices^1^. In biology, ion transport across the cell membrane is vital for regulating physiological functions, such as generating electrical signals in response to external stimuli, secreting hormones and neurotransmitters, and maintaining homeostasis. Activation upon exposure to external stimuli and the resultant selective ion passage through ion channels are fundamental functions of cellular systems to adapt and respond to environmental changes. Mimicking biological ion channels is an efficient approach for designing devices that are responsive to physical or chemical stimuli through an ion-flow-mediated process.

Previously, artificial ion channels have been demonstrated for various external stimuli, such as electrical^2–4^, pH^2^, light^5^, pressure^6^, and chemical^7^ stimuli. Light has been widely utilized as a triggering source for ion gating in nanofluidic circuits owing to its remote controllability, straightforward operation, and diversity of photoactive materials. In particular, numerous types of photoelectric and photothermal materials^8^ facilitate the development of highly efficient solar energy conversion systems for nanochannels. Two-dimensional (2D) nanosheets are advantageous for the development of artificial ion channels owing to their facile fabrication compared with other materials, such as hydrogels^2^, photolithographically patterned Si^9,10^, and charged nanoparticles^11^. To date, various photosensitive artificial ion channels have been proposed with different types of 2D nanosheets^5,12,13^. Specifically, MXenes have the competitive advantages of a rich surface chemistry and a photothermal conversion efficiency of ~100%, with a high extinction coefficient^14^.

**Fig. 1:**
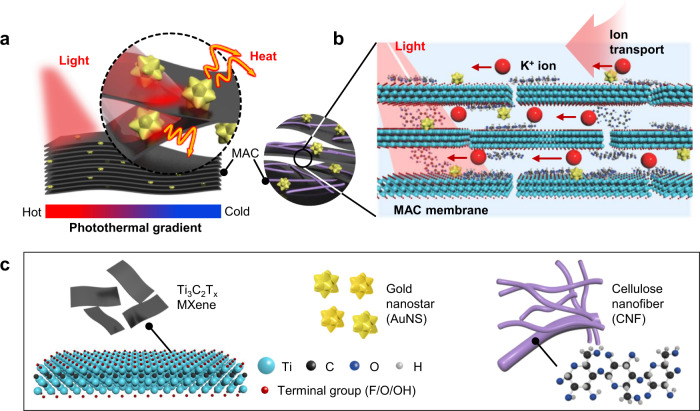
Schematic illustration of photosensitive MXene/AuNS/CNF (MAC) membrane. **a** MAC membrane under exposure to localized NIR light. The plasmonic AuNSs and MXene nanosheets enhance the photothermal effect of the MAC membrane to generate photothermal gradient across the membrane. **b** Directional cation transport through the MAC membrane under photothermal gradient, which is driven by the interfacial photothermo-osmotic flow. **c** Schematic description of MAC components including MXene, AuNS, and CNF.

Herein, we propose a photoresponsive ion channel using 2D MXene (Ti_3_C_2_T_x_) nanosheets, gold nanostars (AuNSs), and cellulose nanofibers (CNFs) to form a MXene/AuNS/CNF (MAC) composite (Fig. 1). The plasmonic AuNSs are intercalated in the layered MXene nanosheets and function as photo-antennae^15^ to absorb light efficiently and transport heat to the adjacent MXene nanosheets. The strong light-to-heat conversion properties of both MXenes and AuNSs arising from their localized surface plasmon resonance (LSPR) effect intensify the light absorbance in the near-infrared (NIR) band. Upon NIR-light illumination, the synergistic plasmonic photothermal effects of MXene and AuNS allow the efficient photothermal activation of the ion channel. Moreover, the CNFs are homogeneously distributed inside the membrane to increase the mechanical integrity and widen the interlayer spacing of the layered MXene structure for an efficient ion flow with less spatial hindrance. Accordingly, the lamellar-structured MAC membrane provides subnanometer channels for ion transport, which induce the overlap of the electric double layer (EDL) on each plane and result in permselective ion transport^16–18^. The MAC offers a selective cationic flow through the nanoconfined space owing to the large number of surface termination groups of MXene and the negatively charged AuNSs and CNFs. Our proposed MAC composite with the coupled plasmonic effects of MXene and AuNS and the CNF nanofibrous spacer exhibits a seven-fold enhanced photothermal current compared with that of the pristine MXene membrane, 40 times higher light-induced current than that of previously reported graphene oxide (GO) membranes^5^, and 14 times higher ionic than that of the GO/AuNS/CNF (GAC) composites. This plasmonic MAC composite enables promising applications in all-solid flexible ion channels and light-driven nanofluidic circuits.

## Results

### Characterization of photosensitive MAC membrane

**Fig. 2:**
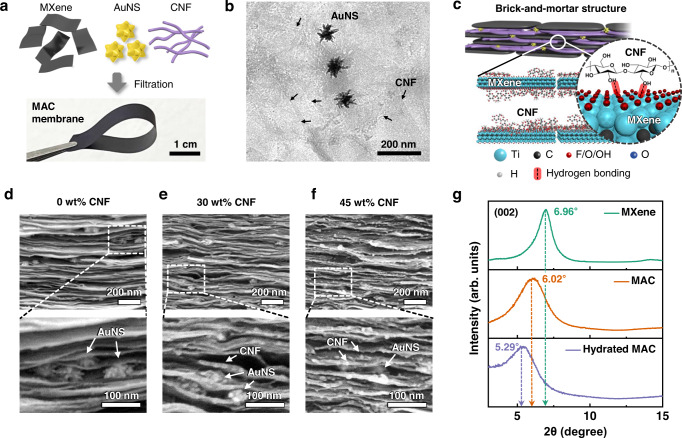
Characterization of the nanoconfined MAC ion channel. **a** Schematic of the MAC components and a photograph of the freestanding MAC film. **b** TEM image of the MAC film. The black arrows indicate CNFs. **c** Schematic of the brick-and-mortar structure of the MAC and the intermolecular interaction between MXene and CNFs. Cross-sectional SEM image of the MAC with increasing amount of CNFs **d** 0 wt%, **e** 30 wt%, and **f** 45 wt% CNFs. **g** XRD spectra of MXene, the MAC, and the hydrated MAC membrane. MXene (20 mg) and MAC (7 wt% of AuNSs and 35 wt% of CNFs in 20 mg of MXene) were utilized for the XRD spectra. The MAC membrane was soaked in DI water overnight for the preparation of the hydrated MAC membrane. Source data are provided as a Source Data file.

The MAC membrane is a photosensitive lamellar-structured ion channel. The MAC components exhibited negative surface charges (Supplementary Fig. 1), which were attributed to a large number of surface functional groups (O, OH, and F) of the MXene sheets, citrate-stabilized AuNSs, and abundant hydroxyl groups in the CNFs. The negatively charged MXene nanosheets, AuNSs, and CNFs were mixed and reassembled via vacuum filtration to prepare a freestanding MAC membrane (Fig. 2a). The MAC components were visually investigated using transmission electron microscopy (TEM, Fig. 2b), where the CNFs and AuNSs were homogeneously dispersed within the stacked layers of the MXene nanosheets. The MAC membrane formed a “brick-and-mortar” structure owing to the CNFs bound to the MXene nanosheets as illustrated in Fig. 2c. The MXene nanosheets (“bricks”) were interconnected by the soft CNFs (“mortar”), which increased the mechanical integrity through a hierarchically organized structure. The brick-and-mortar structure of the MAC membrane was identified from the morphological differences according to the increment in the amount of CNFs in the MAC membrane, as shown in Fig. 2d–f. When the amount of CNFs was increased from 0 to 45 wt%, the MAC membrane had more white-dot-like CNF moieties and a distinguished thicker single lamellar layer originating from the abundant amount of CNFs bound to the MXene sheets through hydrogen bonding. Consequently, the hybridization of CNFs and MXene with the nacre-like structure provides enhanced mechanical strength and flexibility and a higher surface-charge density to increase the hydrophilicity of the membrane^19,20^. Owing to the hydrophilicity and toughening effect of the CNFs, the obtained MAC membrane showed higher hydrophilicity (water contact angle: ~34.7°, Supplementary Fig. 2d) than other materials (MXene, MXene/AuNSs, and MXene/CNFs, Supplementary Fig. 2a–c) and maintained its initial shape in water even after 10 days, demonstrating high aqueous stability (Supplementary Fig. 3). In particular, pristine MXene remained more stable than pristine GO because of the inherently stronger van der Waals attraction and lower electrostatic repulsion between the nanosheets^21^. In addition, the intermolecular hydrogen bonding of MXene and CNFs further enhances the aqueous stability for the development of ion channels in solutions with low salinity.

Moreover, the introduction of charged nanofibrils played a key role in enhancing ion transport by widening the interspacing of the nanochannels. The intercalated CNFs in the MXene film increased the film thickness from 5.4 to 13.4 μm, whereas the MXene/AuNSs film had a similar thickness (5.7 μm) to that of MXene (Supplementary Fig. 4). Meanwhile, the addition of CNFs rarely influences on the electrical conductivity of the MAC film because electrons flow through the interconnected MXene nanosheets in the parallel direction (Supplementary Fig. 5). The prepared MXene nanosheets had a thickness of 1.3 nm and a lateral width of ~0.9 μm (Supplementary Fig. 6). The synthesized nanosheets exhibited a thicker monolayer compared with the theoretical ones (~0.98 nm) owing to the adsorbed water molecules, which is attributed to the hydrophilic nature of the surface functional groups. The enlarged interspacing of the MAC membrane compared with that of pristine MXene can be clarified through X-ray diffraction (XRD) measurements. MXene exhibited a (002) diffraction peak at 2*θ* = 6.96°, which shifted to 6.02° for the MAC membrane, indicating that the interlayer spacing (*d*) had increased from 1.27 to 1.47 nm (Fig. 2g). The augmented d-spacing confirms the homogeneous loading of CNFs into the MXene sheets through hydrogen bonding. In addition, after the MAC membrane was hydrated in water, the (002) diffraction peak of the hydrated MAC membrane was downshifted to 5.29°, indicating a further increase in the d-spacing from 1.47 to 1.67 nm owing to the adsorption of water molecules into the MAC nanosheets. Considering that the theoretical monolayer thickness of MXene is 0.98 nm^22,23^, the hydrated MAC exhibited an effective interlayer spacing of ~0.7 nm for fluid transport, which is larger than the sizes of water molecules and hydrated ions^24^. Therefore, the larger effective interlayer spacing with respect to the diameter of water and hydrated ions allows efficient ion transport through the hydrated MAC nanochannel.

**Fig. 3:**
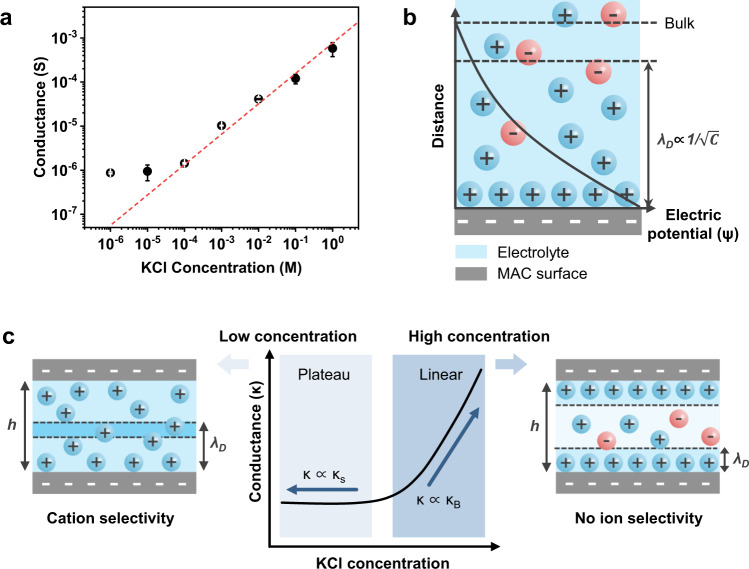
Ion transport through the MAC nanochannel. **a** Ionic conductance of the MAC membrane under different KCl concentrations. **b** Schematic of EDL formation on the negatively charged MAC surface. **c** Ion-transporting behavior under low concentrations of electrolyte (*h*~2*λ*_D_; left) and under high concentrations of electrolyte (*h*»*λ*_D_; right). The ionic conductance (*κ*) is the sum of bulk conductance (*κ*_*B*_) and surface-charge-governed conductance (*κ*_*S*_). *κ*_*S*_ is dominant under a low concentration of electrolyte while *κ*_*B*_ is dominant under a high concentration of electrolyte. Error bars in (**a**) denote standard deviation from three different samples. Source data are provided as a Source Data file.

The transnanochannel ionic conductance strongly depends on the electrolyte concentration. The ionic conductance through the MAC membrane was measured at an equivalent concentration of electrolytes (Supplementary Fig. 7). After the MAC membrane was fully hydrated with KCl, the ion flow across it was observed through ionic current–voltage measurements. Figure 3a shows the conductance of the MAC membrane depending on the KCl concentration. The transnanochannel ionic conductance linearly followed the electrolyte concentration at high concentrations, but became saturated at low concentrations. This behavior is attributed to the competition between the bulk and surface-charge-governed conductances, which is distinguished by the thickness of the EDL. The negatively charged MAC membrane electrostatically interacts with the electrolyte, attracting cations and repelling anions to form an EDL on the channel surface to compensate for the surface charge of the nanochannel. The characteristic thickness of the EDL, Debye screening length (*λ*_D_), is inversely related to the concentration of the electrolyte, which determines the ion selectivity of the nanochannel (Fig. 3b).

The ionic conductance (*κ*) is the sum of the bulk conductance (*κ*_*B*_) and the surface-charge-governed conductance (*κ*_*S*_)^25–27^,

1 $$\kappa={\kappa }_{B}+{\kappa }_{S}=q\left({\mu }_{+}+{\mu }_{-}\right)C{N}_{A}+2\sigma {\mu }_{+}/h,$$

where *q* is the electric charge, *μ*_+_ and *μ*_–_ are the mobilities of the cations and anions, respectively, *C* is the electrolyte concentration, N_A_ is Avogadro’s number, σ is the surface charge of the nanochannel, and *h* is the channel height of the membrane. Under a high concentration of KCl, the bulk conductance (*κ*_*B*_) predominantly affected the ionic conductance because *λ*_D_ was condensed on the surface of the MAC membrane. The shorter *λ*_D_ compared with the channel height (*h* > *λ*_D_) resulted in the coexistence of cations and anions inside the MAC membrane, showing no ion selectivity (Fig. 3c, right). Consequently, at KCl concentrations above 10^–4^ M, the ionic conductance was linearly proportional to the bulk conductance of the electrolyte, according to Eq. (1). Meanwhile, at low KCl concentrations, the surface-charge-governed conductance (*κ*_*S*_) dominantly affected the ion conductance because λ_D_ became comparable to the channel height (*h*~2*λ*_D_), leading to overlapped EDLs and unipolar counterions filling inside the channel. As the MAC membrane carries negative surface charges, the nanochannel was mainly occupied by cations, exhibiting cation permselectivity (Fig. 3c, left). Consequently, at KCl concentrations below 10^–4^ M, the ionic conductance was highly dependent on the surface-charge density of the MAC membrane, resulting in saturated ion conductance, according to Eq. (1). In this work, we utilized the low salinity solution (10^−6^ M of KCl) to achieve cation selectivity and demonstrate directional cation flow under the NIR light.

### Photothermo-osmotic flow through MAC membrane

The high photothermal performance of MXene and AuNSs in the MAC membrane enables efficient NIR-light-responsive ion channels. Compared with other 2D materials (e.g., GO), MXene has a higher photothermal effect owing to its larger extinction coefficient and LSPR effect in the 750–850 nm wavelength range (Supplementary Fig. 8a). In addition, AuNSs provide many hot spots for enhanced local electromagnetic fields and strongly absorb NIR light because of their large molar extinction coefficient and high molar heating rate^28,29^. The AuNSs with sharp surface tips had an LSPR band in the range of 800–900 nm, which was further intensified with the addition of MXene (Supplementary Fig. 8b, c). To identify the photothermal effect of the MAC membrane, we compared the temperature increases of MXene, the MAC membrane, and a polyvinylidene fluoride (PVDF) filter membrane under NIR laser irradiation (Supplementary Fig. 9). The MAC film exhibited a 1.7-fold higher temperature change (ΔT) compared with that of the MXene film owing to the hybridization of plasmonic AuNSs with MXene (Supplementary Fig. 9b).

Under a localized NIR-light stimuli, the MAC membrane can efficiently generate thermal gradient. Specifically, when the photothermally responsive MAC nanochannel is predominantly occupied by cations, it exhibits directional cation flow, which is driven by (photo)thermo-osmosis. Thermo-osmotic flow is fluid transport driven by the temperature gradient in a nonisothermal system. When fluid is confined in a nanospace, hydrophilic fluid tends to interact with the hydrophilic nanochannel and the thermo-osmotic fluid flows along the channel interface. As thermo-osmotic flow is an interfacial flow and the nanoconfined fluid (NCF) is dominantly affected by the surface forces of the nanochannel, the thermo-osmotic transport of the NCF is strongly governed by surface properties, such as hydrophilicity^30^. The thermo-osmotic velocity (**v**_**t**_) can be obtained as follows:

2 $${{{{{{\bf{v}}}}}}}_{{{{{{\bf{t}}}}}}}={\beta }_{12}\frac{\nabla T}{T}=\frac{1}{{\eta }_{{{{{\mathrm{int}}}}}}}{\int }_{0}^{\frac{h}{2}}y\Delta H\left(y\right){dy}\frac{\nabla T}{T},$$

where *β*_12_ is the thermo-osmotic coefficient arising from the thermal gradient $\nabla T$. *η*_*int*_ is the interfacial viscosity, h is the height of the nanochannel, *ΔH(y)* is the excess specific enthalpy compared with that of the bulk, and y is the distance from the channel surface (*y* = 0 indicates the channel surface, and *y* = $\frac{h}{2}$ indicates the center of the channel.). This equation indicates that the transport direction of the NCF is governed by the sign of *β*_12_. The intensity of *β*_12_ is dependent on *η*_*int*_ and the sign of *β*_12_ is determined by the sum of *ΔH(y)* compared with that of the bulk^31,32^. In our study, as the electrolyte was confined by the hydrophilic MAC surface with subnanometer dimensions, it was expected to have a high *η*_*int*_ (no slip flow) and fluctuating excess enthalpy profile with a negative sum of *ΔH(y)*^30–32^. The negative *β*_12_ leads to an NCF transport against the thermal gradient (from the cold to hot sides) according to Eq. (2). Consequently, the directional flow of unipolar cations in the MAC nanochannel induces an ionic current under light stimulus.

**Fig. 4:**
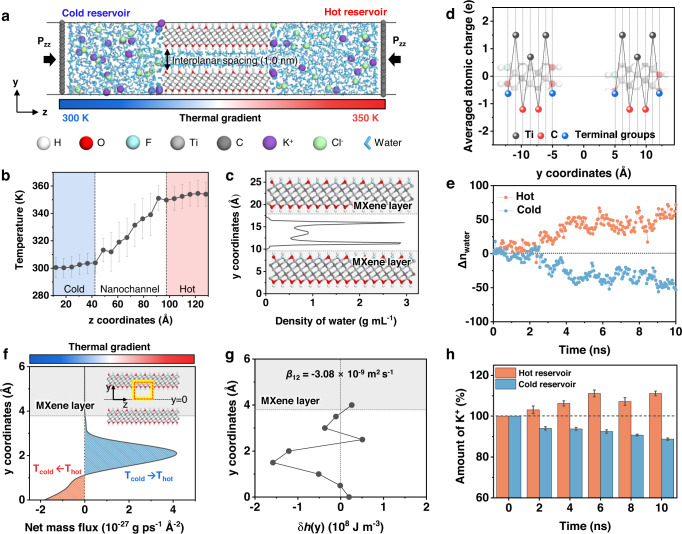
MD simulation of ion flow in the MXene nanochannel. **a** Simulation system of the MXene nanochannel in electrolyte reservoirs at two different temperatures. P_zz_ represents the pressure applied on piston walls along *z* direction. **b** Temperature profile of the solution in the entire system along the direction of the *z* axis, and **c** density profiles of the fluid in the MXene channel along the *y* axis. **d** Averaged atomic charges of the MXene components. The terminal groups of MXene indicate the averaged charges of the O, OH, and F groups. **e** Change in the number of water molecules (Δn_water_) in the cold and hot reservoirs. **f** Net mass flux profile of water in the MXene nanochannel from the center of the channel (*y* = 0) to the wall of the channel. Note that the flux and excess specific enthalpy density at equidistance from the center of the channel (*y* = 0) were averaged. **g** Excess specific enthalpy density of the fluid in the nanochannel. **h** Amount of K^+^ ions in each reservoir with time. Error bars in (**b**) represent the standard deviation for 100 ps during simulation. Error bars in (**h**) denote the standard deviation for 500 ps before each specified time. Source data are provided as a Source Data file.

Molecular dynamics (MD) simulations were performed for pristine MXene-based ion channels to theoretically evaluate the cation selectivity and directional cation transport under a photothermal gradient. The detailed models and methods are summarized in “Methods”. To construct a nonisothermal system and observe ion transport through the pristine MXene channel, MXene nanochannels with an interlayer spacing of 1 nm were placed between cold (*T*_cold_ = 300 K) and hot (*T*_hot_ = 350 K) electrolyte reservoirs (Fig. 4a, “MD simulations” in “Methods”). The temperature difference between the two electrolyte reservoirs (Δ*T* = 50 K) induced a linear thermal gradient through the MXene nanochannel (Fig. 4b), and the nanoconfined water molecules inside the channel were highly assembled near the channel wall because of the strong electrostatic attraction with the terminal groups of MXene (Fig. 4c). Mulliken charge analysis was performed using density functional theory (DFT) calculations, which confirmed the presence of the negatively charged terminal groups of MXenes (i.e., *q*_O_ = −0.82 e, *q*_OH_ = −0.48 e, *q*_F_ = −0.55 e) (Fig. 4d and “DFT calculations” in “Methods”). Owing to the negatively charged MXene surface, cations (i.e., K^+^) were electrostatically attracted to MXene to become closer to the channel wall than anions (i.e., Cl^–^) and were mainly distributed near the O groups of MXene (Supplementary Fig. 10).

In the nonisothermal system, water transport through the nanochannel was observed for 10 ns, where the total water molecules increased in the hot region and decreased in the cold region (Fig. 4e). In this system, the NCF was transported in two different directions, depending on its location inside the nanochannel along the *y* axis (Fig. 4f). When the NCF was at the center of the channel (*y* = 0), it moderately flowed from the hot to cold regions following the thermal gradient, whereas when the NCF was near the wall of the channel, the highly assembled water molecules moved against the thermal gradient (i.e., cold to hot regions) with a high-intensity of net mass flux. We speculated that the flux profile along the channel height showed a trend similar to the thermo-osmotic behavior of the NCF in the hydrophilic channel. Thus, to obtain the thermo-osmotic coefficient in our system, we calculated the excess specific enthalpy density (${{{{{\rm{\delta }}}}}}h\left(y\right)$) in the isothermal state in the MXene channel (see “Excess specific enthalpy calculation” in “Methods”). The sign of the excess specific enthalpy density varied along the *y* axis. At the center of the channel (*y* = 0), the excess specific enthalpy density shows a positive value close to 0, whereas in the region where water is ordered, ${{{{{\rm{\delta }}}}}}h\left(y\right)$ becomes negative owing to the strong binding energy between water and the MXene surface (i.e., the wetting energy of MXene = −0.16 kcal mol^−1^ Å^−2^ from COSMO-DFT calculation, see simulation details in Methods). Note that the sign of *β*_12_ is determined by the integration of excess specific enthalpy, as in Eq. (2). Consequently, the thermo-osmotic coefficient is calculated to be a negative value (i.e., *β*_12_ = −3.08 × 10^–9^ m^2^ s^–1^), theoretically indicating that the water flows against the thermal gradient near the wall (Fig. 4g). The thermo-osmotic coefficient was consistent with the results of our MD simulations for ion transport through the hydrophilic MXene channel. Therefore, we expected that the thermo-osmotic flow exhibited near the MXene interface, transporting NCF against the thermal gradient. Following the fluid transport, the concurrent cation flux was investigated for 10 ns via MD simulations, where directional cation transport was observed from the cold to hot reservoirs (Fig. 4h), whereas most of the chloride ions were stagnant at the reservoirs because they were electrostatically expelled to the original place even if they had entered the MXene channel. The negatively charged MXene channel attracts more cations (i.e., K^+^ ions) near the surface comparing to the anions (i.e., Cl^–^ ions). Therefore, there is a rare chance of the existence of Cl^−^ ions near the channel surface and the net flow of Cl^–^ ions through the MXene channel (Supplementary Fig. 11).

Furthermore, the increment of cations (i.e., K^+^ ions) in the hot reservoir obtained in the simulation was similarly observed in the experimental results shown in Supplementary Fig. 12. The directional cation flow was experimentally investigated using inductively coupled plasma optical emission spectroscopy. The MAC membrane near spot 1 was exposed to NIR light to generate a photothermal gradient across it. The number of K^+^ ions on the hot side (spot 1) increased, whereas that on the cold side (spot 2) decreased compared with the initial concentration (Supplementary Fig. 12b), demonstrating that K^+^ ions flow against the thermal gradient during localized NIR illumination.

The amounts of CNFs and AuNSs in the MAC membrane affect the photothermal current and voltage under the same light intensity. When the amount of CNFs was increased from 0 to 42 wt% with a fixed amount of AuNSs (7 wt%), the MAC membrane showed a maximum photothermal current (Δ*I*) and voltage (Δ*V*) at 35 wt% CNFs (Supplementary Fig. 13a). When the amount of CNFs exceeded 35 wt%, Δ*I* and Δ*V* decreased because of the steric hindrance effect of the nanofibers on the flow of ions through the channel spacing. The increase in the amount of CNFs from 0 wt% to 42 wt% led to an increase in Δ*T* (3.8 K) from 42.9 K to 46.7 K. This behavior is attributed to the low thermal conductivity (*k*) of CNFs, which results in decreased heat transfer from the MAC film to the ambient air. Compared with the high *k* value of metallic MXene^33^, CNF has a 40 times lower *k*^34^, resulting in lower heat dissipation and, thus, a higher Δ*T*. Furthermore, intertwined CNF-bound MXene composites can increase the internal reflection of thermal radiation and enhance the photothermal effect^35^. The complex interfaces between CNF and MXene lower the heat transfer from the solid to the outer space by multiple phonon scattering events in the inner space of the MAC film.

In addition, the effect of AuNSs on the photothermal current and voltage was examined under the same amount of CNFs (35 wt%) (Supplementary Fig. 13b). It was observed that 7 wt% AuNSs demonstrated the best photothermal performance. Although the influence of AuNSs on the photothermal current/voltage behavior was minor compared with the effect of CNFs, they increased the photothermal effect with the increase in light–matter interaction under the same light intensity, resulting in an increase in ΔT (~2.1 K) from 44.4 to 46.5 K. The optimized MAC ratio (7 wt% of AuNSs and 35 wt% of CNFs in 20 mg of MXene dispersion) was utilized for further experiments to observe the photothermal current under various conditions, unless otherwise specified.

### Photothermal ionic current of MAC membrane

**Fig. 5:**
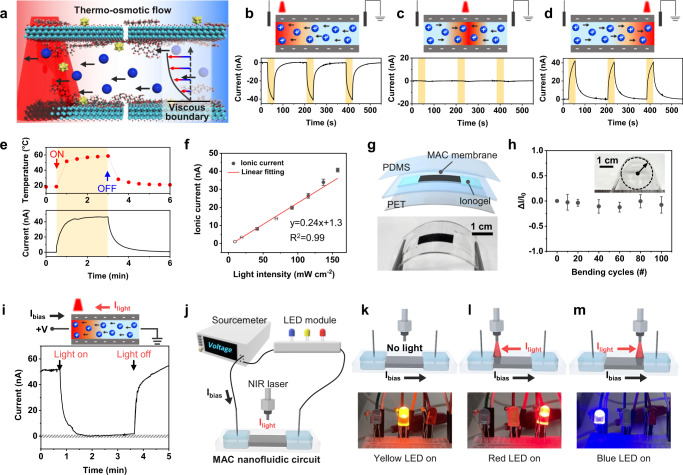
Photothermal ionic current of the MAC membrane. **a** Schematic of thermo-osmotic flow in the nonisothermal system. Ionic current of the MAC membrane depending on the location of light irradiation: **b** left, **c** center, and **d** right. **e** Saturated ionic current under continuous NIR irradiation. **f** Linearly fitted photothermal current under different light intensities. **g** Schematic and photograph of the flexible ionogel–MAC film. **h** Relative current changes (ΔI/I_0_) of the ionogel–MAC film under 100 bending cycles. Inset photograph indicates a bent ionogel–MAC sample with a bending radius of 1.2 cm. **i** Photothermally induced ionic switch under a constant voltage bias of 45 mV. **j** Proof-of-concept demonstration of the MAC-based nanofluidic circuit with LED modules showing different LED bulbs turning on depending on the site of light exposure. Two voltage thresholds for Arduino are set to switch LEDs (*V*_high_ = 65 mV, *V*_low_ = 25 mV). The Arduino is set to light (**k**) yellow LED (*V*_low_ <*V* < *V*_high_), **l** red LED (*V* < *V*_low_), and **m** blue LED (V > *V*_high_). All the MAC samples are prepared with the optimized MAC ratio (7 wt% AuNSs, 35 wt% CNFs, 20 mg MXene) and soaked in 1 μM KCl electrolyte. Panels **b**–**e**, **h**, **i**, **k**–**m** are obtained under the same light intensity (157 mW cm^–2^). Error bars in (**f**, **h**) denote standard deviation from three different samples. Source data are provided as a Source Data file.

The (photo)thermo-osmotic flow induced a directional ionic current through the MAC film toward the region under NIR-light exposure (Fig. 5a). Thus, the direction and intensity of the ionic current can be controlled by the light-irradiated position of the MAC film (Fig. 5b–d). When the left or right edge of the membrane was exposed to NIR light, cations flowed against the thermal gradient and induced a negative or positive current but with the same intensity (Fig. 5b, d). However, when the center of the MAC membrane was exposed to NIR light, cations flowed from both ends of the channel to the center of the membrane, leading to an unnoticeable ionic current (Fig. 5c). Meanwhile, pristine MXene underwent the same directional cation flow but with lower current and voltage (Supplementary Fig. 14), indicating that the AuNSs and CNFs in the MAC membrane are critical for the efficient flow of cations through the MAC nanochannel. The MAC membrane has approximately seven times higher photothermal current and ~1.6 times higher photothermal voltage compared with those of the pristine MXene channel under the same NIR-light intensity. Similar to MXene, GO nanosheets with oxygen-containing groups on the surface can be utilized as hydrophilic and negatively charged lamellar-structured ion channels. Compared to the pristine GO membrane, intermolecular bonding between GO nanosheets and CNFs leads to the better aqueous stability of a GO/AuNS/CNF (GAC) membrane (Supplementary Fig. 15a, b). GAC membrane also exhibits cation selectivity but showed 14 times lower ionic current than that of the MAC membrane (Supplementary Fig. 15c, d). The higher ionic current of MAC comparing to the GAC membrane is attributed to the abundant surface functional groups of MXene and great photothermal conversion effect of MXene-based composites. Therefore, MAC composite showed 1.7 times higher Δ*T* than GAC composite (45 K for MAC and 27 K for GAC) due to the MXene’s large extinction coefficient and LSPR effect in the NIR range.

The photothermally responsive MAC membrane also exhibited a continuous ionic current under long-term irradiation with NIR light. After exposure of the edge of the MAC membrane to NIR light for 2.5 min, the temperature and current became saturated (Fig. 5e). Figure 5f and Supplementary Fig. 16 demonstrate the linear dependence of the photothermal current on the NIR-light intensity. The ionic current linearly increased with the NIR-light intensity from 9.4 to 157.6 mW cm^–2^. In addition, as the photothermal gradient was the driving force of the ionic current across the MAC membrane, the photothermally induced temperature change (Δ*T*) showed a linear relationship with the ionic current (Supplementary Fig. 17). The ionic currents and voltages through various 2D-material-based nanochannels are summarized in Supplementary Table 2. The MAC membrane exhibited ionic current and voltage values comparable to or higher than those of the GO-based membrane and pristine MXene. Furthermore, few studies have explored thermo-osmotic flow through 2D-material-based nanochannels. The MAC nanochannel shows a highly stable current over 11 cycles of light being switched on and off, maintaining 92.5% of the initial ionic current value (Supplementary Fig. 18). Furthermore, ionic current changes (*I*/*I*_0_) were measured for 12 days to investigate the long-term stability of MAC membrane (Supplementary Fig. 19). The ionic current maintained 92% of initial current over a week owing to the restrained oxidation of the MAC membrane encapsulated in elastomer and the exposure to the electrolyte reservoirs and NIR-light irradiance only on the localized area.

In addition, when an ionic hydrogel is used as the electrolyte, the MAC membrane can be used as a flexible ion channel. The flexible ionogel–MAC sample was prepared by encapsulating an ionic polyacrylamide (PAM) hydrogel and the MAC membrane between bottom polyethylene terephthalate (PET) and top polydimethylsiloxane (PDMS) layers (Fig. 5g). When the ionogel–MAC sample was exposed to localized NIR light with different light intensities, the ionic current increased with light intensity and exhibited a linear correlation with the light intensity (Supplementary Fig. 20). For repeated bending cycles at a bending radius of 1.2 cm, the ionogel–MAC sample retained stable relative ionic current values (Δ*I*/*I*_0_; *I*_0_ is the ionic current before bending) during 100 bending cycles (Fig. 5h). The bending test results suggest that the ionogel–MAC sample could be potentially utilized for portable and wearable photoresponsive devices, maintaining its performance under repeated deformation.

The light-responsive MAC channel can be exploited as a nanofluidic circuit to switch off or amplify current depending on the light-exposed sites. As shown in Fig. 5i, the MAC membrane was utilized as an ionic photo switch, where the light-driven ion flow counterbalanced the ion flow induced by the voltage bias. Under a constant voltage bias (45 mV), the external voltage drags ions, resulting in an ionic current of ~50 nA across the nanochannel. When NIR light is incident onto the MAC membrane, the light-driven reverse ionic current (*I*_light_) counterbalances the bias-driven ionic current (*I*_bias_). Consequently, the light-driven ionic current induces the nanofluidic circuit to switch off with an on/off ratio of up to 10^4^. As a proof-of-concept demonstration, we connected a sourcemeter, the MAC channel, and an LED module to test the MAC nanofluidic circuit for switching light bulbs (Fig. 5j and Supplementary Fig. 21) depending on two voltage thresholds (65 mV and 25 mV). The light-tunable ionic current (*I*_light_) can modulate the voltage, which, in turn, switches the LED bulbs (Fig. 5k–m and Supplementary Fig. 22). At the initial stage, a yellow LED was turned on, indicating that only *I*_bias_ flowed (Fig. 5k). When NIR light was incident onto the left side of the MAC channel, *I*_light_ was induced, which reduced *I*_bias_ and turned the red LED on (Fig. 5l and Supplementary Movie 1). Meanwhile, when the opposite side of the MAC channel was illuminated, *I*_light_ was in the same direction as *I*_bias_, which amplified the total ionic current and turned the blue LED on (Fig. 5m and Supplementary Movie 2). Consequently, the diminishing or amplifying current through the circuit can be visualized from simple LED lights owing to the light-tunable ionic current through the MAC channel.

## Discussion

In summary, we demonstrated a photothermally responsive MAC membrane containing plasmonic AuNSs and CNFs in MXene nanosheets. In this structure, AuNSs were intercalated in the layered MXene nanosheets to enhance the photothermal effect of the MAC composites. In addition, CNFs distributed inside the membrane improved the mechanical integrity and increased the interlayer spacing for facile ion flow with reduced spatial hindrance. Accordingly, the nano-spaced, negatively charged, and hydrophilic MAC membrane electrostatically attracted counterions (i.e., cations) and the nanoconfined fluid tended to be localized near the channel wall with high mass density, which was directionally transported through the MAC membrane under the light stimulus. When NIR light was locally irradiated on the MAC membrane, thermal gradient was formed across the MAC nanochannel, which resulted in directional cationic flow driven by interfacial photothermo-osmotic flow. The optimized MAC membrane showed photothermal ionic current of ~40 nA that was nearly seven times higher than that of the pristine MXene-based nanochannel and 14 times higher ionic current than that of the GAC membrane. An all-solid flexible MAC film could be used as a flexible photoresponsive ion channel through the incorporation of a PAM-based ionogel. Furthermore, the light-responsive MAC membrane can be potentially utilized in nanofluidic circuits, such as ionic photoswitches.

## Methods

### Materials

Gold (III) chloride solution (HAuCl_4_, 99.99% trace metals basis), sodium citrate (≥99.0%), l-ascorbic acid (≥99.0%), and silver nitrate (AgNO_3_, ≥99.0%) were purchased from Sigma-Aldrich for synthesizing AuNSs. The MAX precursor Ti_3_AlC_2_ (11 technology, China), Lithium fluoride (LiF, >98.5%, Alfa Aesar), and Hydrochloric acid (HCl, 35%, Daejung, South Korea) were used for preparing MXene (Ti_3_C_2_T_x_). The CNFs were purchased from ANPOLY (South Korea). Graphite powder (SP-1, Bay Carbon), potassium peroxodisulfate (K_2_S_2_O_8_, ≥99.0%, Sigma), phosphorus pentoxide (P_2_O_5_, ≥98.0%, Sigma), potassium permanganate (KMnO_4_, ≥99.0%, Sigma), hydrogen peroxide (H_2_O_2_, 35%, Duksan, South Korea), and sulfuric acid (H_2_SO_4_, 95.0%, Daejung, South Korea) were used for preparing GO. All the chemicals were utilized without further purification.

### Synthesis of Ti_3_C_2_T_x_ MXene nanosheets

MXene was synthesized using a minimally intensive layer-delamination method^36^. This method involves selective etching of Al layers from the MAX precursor Ti_3_AlC_2_. LiF (3.2 g) was dissolved in HCl (9 M, 40 mL) under continuous stirring for 20 min. Then, the MAX powder (2 g) was gradually added to the etchant, and the solution was moderately stirred for 24 h at 35 °C. The solution was washed repeatedly with deionized (DI) water by centrifugation (1500×*g* for 5 min). When the pH was neutralized to approximately 6, the centrifugation time was increased from 5 min to 1 h. The settled Ti_3_C_2_T_x_ sediment was collected and sonicated for 1 h in an ice bath to delaminate Ti_3_C_2_T_x_ flakes. The mixture was then centrifuged at 1500×*g* for 1 h. The supernatant obtained was collected, vacuum-filtered, and stored under a vacuum.

### Synthesis of graphene oxide nanosheets

Graphene oxide was synthesized through a modified Hummers method. In brief, graphite powder, K_2_S_2_O_8_, and P_2_O_5_ were dispersed in concentrated H_2_SO_4_ solution, which was stirred for 4.5 h at 80 °C. Then, this solution was diluted in DI water and filtrated with DI washing for pH neutralization. The filtrated one was dried in a vacuum condition overnight. The pre-oxidized graphite was then dispersed in concentrated H_2_SO_4_. Then, KMnO_4_ was gently added to the solution under vigorous stirring, followed by stirring at 36 °C for 2 h. The mixture was gradually diluted with DI water and further stirred for 2 h. Then, 30% H_2_O_2_ was added to this mixture, which resulted in the color change to bright yellow with violent bubbles. Subsequently, this mixture was stirred for 1 h, centrifuged several times with the diluted HCl, and the resulting mixture was subjected to dialysis to adjust the acidity. Finally, the resulting solution was concentrated by drying in a 30 °C vacuum condition and redispersed in DI water by sonication to obtain aqueous GO solution.

### Synthesis of gold nanoparticle seed (AuNP) and gold nanostars (AuNS)

AuNSs were synthesized via AuNP-seed-mediated growth. The AuNPs and AuNSs were prepared according to methods described in the literature^37^. For the synthesis of AuNP seeds with an LSPR of approximately 519 nm, 50 mL of DI water was poured into a two-neck round bottom flask and placed in an oil bath at a temperature of 105 °C with vigorous stirring. When the temperature was stabilized, 5 μL of HAuCl_4_ and 1 mL of sodium citrate solution (0.01 mg mL^–1^) were sequentially added to the flask. After approximately 15–20 min, the color of the solution changed from transparent to grayish purple, and the final AuNP-seed solution showed a ruby red color. The final product was cooled in cold water and stored at 4 °C for AuNS growth.

For the synthesis of AuNSs with an LSPR in the NIR region, 10 mL of 0.25 mM HAuCl_4_ was added to a glass vial and stirred at 1500 rpm. After adding 800 μL of the previously synthesized AuNP-seed solution, sequential addition of HCl, ascorbic acid, AgNO_3_, and sodium citrate solution was required. First, 10 μL of 1 M HCl solution was added to lower the pH. Second, 50 μL of 0.1 M ascorbic acid and 100 μL of 10 mM AgNO_3_ were added quickly at the same time to observe the change in the color of the solution to dark bluish-gray. Finally, after stirring for 30 s, 200 μL of 100 mM sodium citrate was added as a capping agent and stirred for another 10 s. For further sampling of the MXene/CNF dispersion, we concentrated the as-prepared AuNS solution via centrifugation at 1500×*g* for 15 min.

### Preparation of MAC composite film

The Ti_3_C_2_T_x_ nanosheets were redispersed in DI water (1 mg mL^–1^) and mixed with a diluted CNF dispersion (0.1 wt% in DI water) and a 10-fold concentrated AuNS solution, sequentially. The mixture was vortexed and sonicated for 15 min to obtain a homogeneous MAC dispersion. The mixture was vacuum-filtered using a hydrophilic PVDF filter membrane and washed with a large amount of DI water to remove the citrate crystals. After drying the filtered membrane, the film was dried under a vacuum for a day and then peeled off from the supporting membrane.

### MD simulations

Molecular dynamics (MD) simulations were performed with LAMMPS^38^. We built a model system of the MXene (Ti_3_C_2_T_x_) channel, which is connected to two 1 M KCl solution reservoirs and two rigid graphene pistons to apply constant pressure (*P* = 1 atm) to both sides of the reservoirs in a box dimension of 94.96 × 27.48 × 146.39 Å^3^. The periodic boundary condition was applied for *xyz*-direction of lattice. To prevent periodic interaction between solutions beyond the pistons which restrict the passage of molecules, the periodic distance between pistons along the *z* axis was set as >2.3 nm to secure sufficient volume of vacuum in the *z*-axis direction during the simulation. MXene channels with interplanar spacing of 1.0 nm were energetically relaxed by DFT calculation and the vacant region of the channel was filled with pure water in the initial state of the simulation. All the interactions of the MXene channel and KCl solution were described by the Lennard–Jones equation and the potential parameter and atomic charges are listed in Supplementary Table 1. The cutoff distance was set as 9.0 Å for short-range van der Waals interactions and using the PPPM method^39^ for long-range Coulomb interactions. Note that atomic configurations of MXene were constrained during MD simulation. After relaxing the configuration of the solution system under *NVT* ensemble for 1 ns with Nose–Hoover thermostat^40^ (i.e., *T* = 325 K, decay constant = 0.1 ps), MD simulations for observing ion transport through the nanochannel were performed for 10 ns. The *NVE* ensemble was applied for all atoms, including the piston walls. Simultaneously, the temperature of both reservoirs were adjusted via a canonical velocity-rescaling method^41^. In this method, the temperature of solution in each reservoir was adjusted in a time span of 10 ps to generate thermal gradient in the nanochannel.

### DFT calculations

The Dmol^3^ program was employed to calculate the charge state and wetting energy of the MXene surface^42,43^. The terminating group of MXene model from the work of Cheng et al.^44^ was modified as F: OH: O = 0.3125:0.3125:0.375^45^ to mimic the experimental state. The energetically minimized configuration of the MXene surface model is obtained by quantum mechanical calculations, which were performed using the local density approximation with the Perdew–Wang (LDA-PWC) functional^46^. The convergence tolerance was set as 1.0 × 10^−5^ Ha for energy and 0.002 Ha Å^−1^ for force. A double numerical basis set with polarization functions was used, with a real-space cutoff of 4.4 Å. The calculation method for the wetting energy was based on the work of Delley et al.^47^ with a conductor-like screening model (COSMO)^48^.

### Excess specific enthalpy calculation

To calculate the specific enthalpy in the MXene channel with respect to volume, we conducted an additional MD simulation for 5 ns at 325 K using the same simulation model for the ion-transport system without a thermal gradient. The excess specific enthalpy can be obtained as^49^

3 $${{{{{\rm{\delta }}}}}}h\left(y\right)=h\left(y\right)-{h}_{b},$$

where *h*(*y*) is the local specific enthalpy of water, and *h*_*b*_ is the specific enthalpy of bulk water. The equation for the local specific enthalpy of water in the channel is given by^50^

4 $$h\left(y\right)=u\left(y\right)+{p}_{{zz}}\left(y\right),$$

where *u*(*y*) is the internal energy, which is the sum of the kinetic and potential energies of a molecule per unit volume, and *p*_zz_(*y*) is the pressure of water in the longitudinal direction of the channel. To calculate *u*(*y*) and *p*_zz_(*y*), we divided the channel region using a bin having the size of 94.96 × 0.5 × 0.5 Å^3^, and averaged the internal energy and pressure of the bins located on the same *y* axis. For *h*_*b*_, the additional MD simulations with the enlarged MXene nanochannel of *L* = 4 nm to secure the region of bulk water were conducted and *h*_*b*_ was derived as −0.06 × 10^9^ J m^–3^.

### Characterization

The morphology of the MAC membrane was examined using field-emission scanning electron microscopy (S-4800, Hitachi) and transmission electron microscopy (JEM-2100, JEOL). The d-spacing values of MXene and the MAC membranes were observed using high-power X-ray diffraction (D/MAX2500V/PC, Rigaku). The current–voltage curves of the MAC membrane were obtained using a semiconductor characterization system (4200, Keithley Instruments), and the current and voltage under NIR-light illumination were measured using a sourcemeter (2450, Keithley Instruments). The NIR laser was an 808 nm single-wavelength light source (MDL-F-808 nm, CNI), and the photothermal temperature changes were recorded using an IR camera (Therm-App TH, Therm-App). The surface charges of MXene, AuNS, and CNF were measured using a Zetasizer (Nano ZS, Malvern). The contact angle of the freestanding MXene-based membranes was determined using a contact angle apparatus (Phoenix 300, SEO). Dimensional atomic force microscopy (AFM, Dimension ICON, Bruker Nano Surfaces) was used to characterize the dimensions and thicknesses of the MXene nanosheets. The UV–Vis absorbances of MXene, GO, and AuNSs were measured using a UV–Vis–NIR spectrophotometer (Cary 5000, Agilent). The amount of K^+^ ions after illuminating the MAC membrane with NIR light was determined using inductively coupled plasma optical emission spectroscopy (700-ES, Varian). For the photothermally tunable ionic switch, three different colored light bulbs were serially connected to the circuit.

### Reporting summary

Further information on research design is available in the Nature Portfolio Reporting Summary linked to this article.

## Supplementary information

Supplementary Information

Description of Additional Supplementary Files

Supplementary Movie 1

Supplementary Movie 2

Reporting Summary

## Data Availability

All data are available within the article and its Supplementary Information files. Additional data related to this paper may be requested from the corresponding authors. Source data are provided with this paper.

## Source data

Source Data

## References

[1] Xiao K, Wan C, Jiang L, Chen X, Antonietti M (2020). Bioinspired ionic sensory systems: the successor of electronics. Adv. Mater..

[2] Bao B (2017). 3D porous hydrogel/conducting polymer heterogeneous membranes with electro‐/pH‐modulated ionic rectification. Adv. Mater..

[3] Zhang Q (2018). Highly efficient gating of electrically actuated nanochannels for pulsatile drug delivery stemming from a reversible wettability switch. Adv. Mater..

[4] Rabinowitz J, Cohen C, Shepard KL (2019). An electrically actuated, carbon-nanotube-based biomimetic ion pump. Nano Lett..

[5] Yang J (2019). Photo-induced ultrafast active ion transport through graphene oxide membranes. Nat. Commun..

[6] Chun K-Y, Son YJ, Han C-S (2016). Highly sensitive and patchable pressure sensors mimicking ion-channel-engaged sensory organs. ACS Nano.

[7] Cai S-L (2015). Surface charge modulated aptasensor in a single glass conical nanopore. Biosens. Bioelectron..

[8] Zhu L, Gao M, Peh CKN, Ho GW (2018). Solar-driven photothermal nanostructured materials designs and prerequisites for evaporation and catalysis applications. Mater. Horiz..

[9] Karnik R (2005). Electrostatic control of ions and molecules in nanofluidic transistors. Nano Lett..

[10] Karnik R, Duan C, Castelino K, Daiguji H, Majumdar A (2007). Rectification of ionic current in a nanofluidic diode. Nano Lett..

[11] Lei Y, Wang W, Wu W, Li Z (2010). Nanofluidic diode in a suspended nanoparticle crystal. Appl. Phys. Lett..

[12] Jia P (2021). Harnessing ionic power from equilibrium electrolyte solution via photoinduced active ion transport through van‐der‐Waals‐like heterostructures. Adv. Mater..

[13] Hong S (2020). Photothermoelectric response of Ti_3_C_2_T_x_ MXene confined ion channels. ACS Nano.

[14] Li R, Zhang L, Shi L, Wang P (2017). MXene Ti_3_C_2_: an effective 2D light-to-heat conversion material. ACS Nano.

[15] Lee J (2014). Tailoring surface plasmons of high-density gold nanostar assemblies on metal films for surface-enhanced Raman spectroscopy. Nanoscale.

[16] Sparreboom W, van den Berg A, Eijkel JC (2009). Principles and applications of nanofluidic transport. Nat. Nanotechnol..

[17] Daiguji H (2010). Ion transport in nanofluidic channels. Chem. Soc. Rev..

[18] Huang X, Kong XY, Wen L, Jiang L (2018). Bioinspired ionic diodes: from unipolar to bipolar. Adv. Funct. Mater..

[19] Cao W-T (2018). Binary strengthening and toughening of MXene/cellulose nanofiber composite paper with nacre-inspired structure and superior electromagnetic interference shielding properties. ACS Nano.

[20] Zeng Z (2020). Nanocellulose‐MXene biomimetic aerogels with orientation‐tunable electromagnetic interference shielding performance. Adv. Sci..

[21] Lao J (2018). Aqueous stable Ti_3_C_2_ MXene membrane with fast and photoswitchable nanofluidic transport. ACS Nano.

[22] Wang X (2015). Atomic-scale recognition of surface structure and intercalation mechanism of Ti3C2X. J. Am. Chem. Soc..

[23] Ghidiu M, Lukatskaya MR, Zhao M-Q, Gogotsi Y, Barsoum MW (2014). Conductive two-dimensional titanium carbide ‘clay’with high volumetric capacitance. Nature.

[24] Wang L (2017). Fundamental transport mechanisms, fabrication and potential applications of nanoporous atomically thin membranes. Nat. Nanotechnol..

[25] Schoch RB, Renaud P (2005). Ion transport through nanoslits dominated by the effective surface charge. Appl. Phys. Lett..

[26] Raidongia K, Huang J (2012). Nanofluidic ion transport through reconstructed layered materials. J. Am. Chem. Soc..

[27] Li T (2019). A nanofluidic ion regulation membrane with aligned cellulose nanofibers. Sci. Adv..

[28] Wang X, Li G, Ding Y, Sun S (2014). Understanding the photothermal effect of gold nanostars and nanorods for biomedical applications. RSC Adv..

[29] Freddi S (2013). A molecular thermometer for nanoparticles for optical hyperthermia. Nano Lett..

[30] Sun C, Zhou R, Zhao Z, Bai B (2020). Nanoconfined fluids: what can we expect from them?. J. Phys. Chem. Lett..

[31] Wang X, Liu M, Jing D, Mohamad A, Prezhdo O (2020). Net unidirectional fluid transport in locally heated nanochannel by thermo-osmosis. Nano Lett..

[32] Fu L, Merabia S, Joly L (2017). What controls thermo-osmosis? Molecular simulations show the critical role of interfacial hydrodynamics. Phys. Rev. Lett..

[33] Liu R, Li W (2018). High-thermal-stability and high-thermal-conductivity Ti3C2T x MXene/poly (vinyl alcohol)(PVA) composites. ACS Omega.

[34] Yang W (2017). Ultrathin flexible reduced graphene oxide/cellulose nanofiber composite films with strongly anisotropic thermal conductivity and efficient electromagnetic interference shielding. J. Mater. Chem. C..

[35] Cui Y, Gong H, Wang Y, Li D, Bai H (2018). A thermally insulating textile inspired by polar bear hair. Adv. Mater..

[36] Alhabeb M (2017). Guidelines for synthesis and processing of two-dimensional titanium carbide (Ti3C2T x MXene). Chem. Mater..

[37] Yuan H (2012). Gold nanostars: surfactant-free synthesis, 3D modelling, and two-photon photoluminescence imaging. Nanotechnology.

[38] Plimpton S (1995). Fast parallel algorithms for short-range molecular dynamics. J. Comput. Phys..

[39] Hockney, R. W. & Eastwood, J. W. *Computer Simulation Using Particles* (CRC Press, 2021).

[40] Hoover WG (1985). Canonical dynamics: equilibrium phase-space distributions. Phys. Rev. A.

[41] Bussi G, Donadio D, Parrinello M (2007). Canonical sampling through velocity rescaling. J. Chem. Phys..

[42] Delley B (1990). An all‐electron numerical method for solving the local density functional for polyatomic molecules. J. Chem. Phys..

[43] Delley B (2000). From molecules to solids with the DMol 3 approach. J. Chem. Phys..

[44] Cheng R (2018). Understanding the lithium storage mechanism of Ti3C2T x MXene. J. Phys. Chem. C..

[45] Hart JL (2019). Control of MXenes’ electronic properties through termination and intercalation. Nat. Commun..

[46] Perdew JP, Wang Y (2018). Erratum: accurate and simple analytic representation of the electron-gas correlation energy [Phys. Rev. B 45, 13244 (1992)]. Phys. Rev. B.

[47] Delley B (2006). The conductor-like screening model for polymers and surfaces. Mol. Simul..

[48] Klamt, A. & Schüürmann, G. COSMO: a new approach to dielectric screening in solvents with explicit expressions for the screening energy and its gradient. *J. Chem. Soc. Perkin Trans.* **2**, 799–805 (1993).

[49] Denbigh KG, Raumann G (1952). The thermo-osmosis of gases through a membrane. II. Experimental. Proc. R. Soc. Lond. Ser. A. Math. Phys. Sci..

[50] Ganti R, Liu Y, Frenkel D (2017). Molecular simulation of thermo-osmotic slip. Phys. Rev. Lett..

