# Supplementary material for: Photosensitive ion channels in layered MXene membranes modified with plasmonic gold nanostars and cellulose nanofibers
Source: Nat Commun. 2023 Jan 23;14:359. doi: 10.1038/s41467-023-36039-5 (PMC9870870; doi:10.1038/s41467-023-36039-5)
Supplement: Supplementary file 1 — Supplementary Information [file 41467_2023_36039_MOESM1_ESM.pdf]

Supplementary Information

Photosensitive ion channels in layered MXene membranes modified with plasmonic gold nanostars and cellulose nanofibers

Jeonghee Yeom[1], Ayoung Choe[1], Jiyun Lee[1], Jeeyoon Kim[1], Jinyoung Kim[1], Seung Hak Oh[1], Cheolhong Park[1], Sangyun Na[1], Young-Eun Shin[1], Youngoh Lee[1], Yun Goo Ro[1], Sang Kyu Kwak[2,*] and Hyunhyub Ko[1,*]

[1]School of Energy and Chemical Engineering, Ulsan National Institute of Science and Technology (UNIST), Ulsan Metropolitan City, 44919, Republic of Korea.

[2]Department of Chemical and Biological Engineering, Korea University, 145 Anam-ro, Seongbuk-gu, Seoul, 02841, Republic of Korea

These authors contributed equally: Jeonghee Yeom, Ayoung Choe, Jiyun Lee

* Corresponding author: skkwak@korea.ac.kr (S. K. Kwak), hyunhko@unist.ac.kr (H. Ko)

This PDF file includes:

Supplementary Figures 1-22

Supplementary Tables 1, 2

Supplementary References

Table of contents

Supplementary Fig. 1: Zeta potential of MXene, AuNS, and CNF dispersions.

Supplementary Fig. 2: Water contact angles (WCAs) of the membranes.

Supplementary Fig. 3: Aqueous stability of the membrane.

Supplementary Fig. 4: Cross-sectional SEM images of MXene film, MXene/AuNS, and MAC membrane.

Supplementary Fig. 5: Sheet resistance of MXene and MAC film.

Supplementary Fig. 6: Characterization of the MXene nanosheets.

Supplementary Fig. 7: Schematic of the experimental setup for the observation of ion transport through the MAC membrane.

Supplementary Fig. 8: Photothermal properties of MXene and AuNS.

Supplementary Fig. 9: Photothermal effect of MXene and the MAC membrane.

Supplementary Fig. 10: Ion distribution in the MXene nanochannel.

Supplementary Fig. 11: MD simulation of ion flow in the MXene nanochannel.

Supplementary Fig. 12: Inductively coupled plasma-optical emission spectrometry (ICP-OES) measurement.

Supplementary Fig. 13: Photothermally induced current and voltage with different contents of CNFs and AuNSs.

Supplementary Fig. 14: Photothermally induced current and voltage of the pristine MXene channel.

Supplementary Fig. 15: Aqueous stability and ionic current of GAC.

Supplementary Fig. 16: Photothermally induced ionic current of the MAC channel.

Supplementary Fig. 17: Photothermally induced ionic current of the MAC membrane under temperature changes.

Supplementary Fig. 18: Cyclic ionic current of the MAC membrane.

Supplementary Fig. 19: Long-term stability of MAC ion channel.

Supplementary Fig. 20: Photothermally induced ionic current of the ionogel–MAC channel.

Supplementary Fig. 21: Experimental set-up for LED light switching.

Supplementary Fig. 22: Ionic current under continuous voltage bias.

Supplementary Table 1: Lennard–Jones parameters for the KCl solution and MXene channel. Note that the references indicate the force field used in this study.

Supplementary Table 2: Recent studies on the photo(thermo)-responsive ionic current and voltage through a nanoconfined ion channel.

Supplementary References.

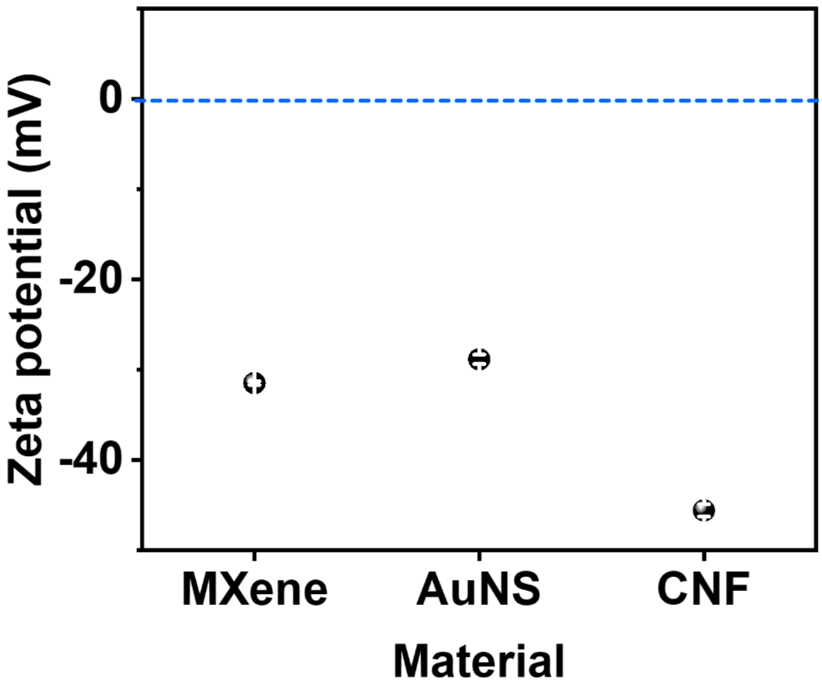

Supplementary Fig. 1. Zeta potential of MXene, AuNS, and CNF dispersions. MXene (0.1 wt%), AuNS (as synthesized), and CNF (0.1 wt%) dispersions were prepared for zeta potential analysis. Error bars represent standard deviation from three measurements (n=3). Source data are provided as a Source Data file.

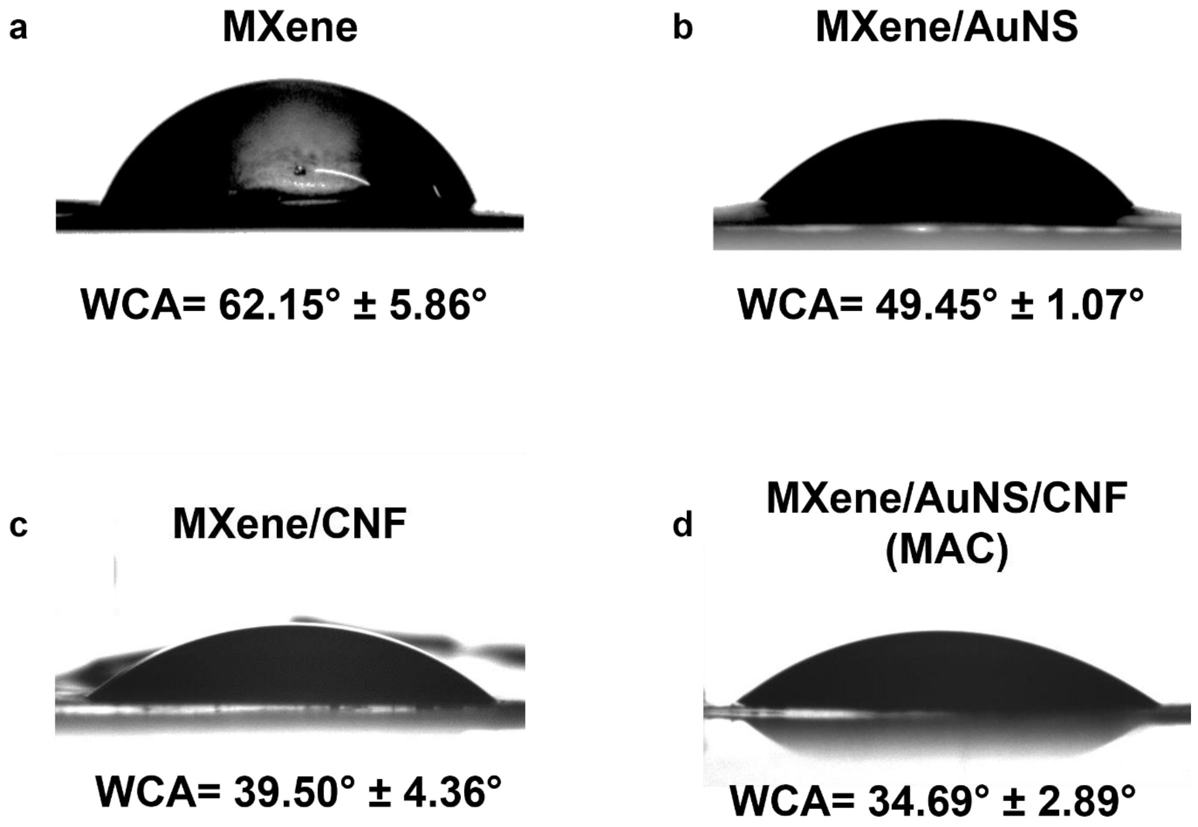

Supplementary Fig. 2. Water contact angles (WCAs) of the membranes. (a) MXene, (b) MXene/AuNS, (c) MXene/CNF, and (d) MXene/AuNS/CNF (MAC) membrane. The WCA values represent the mean and standard deviation from three samples.

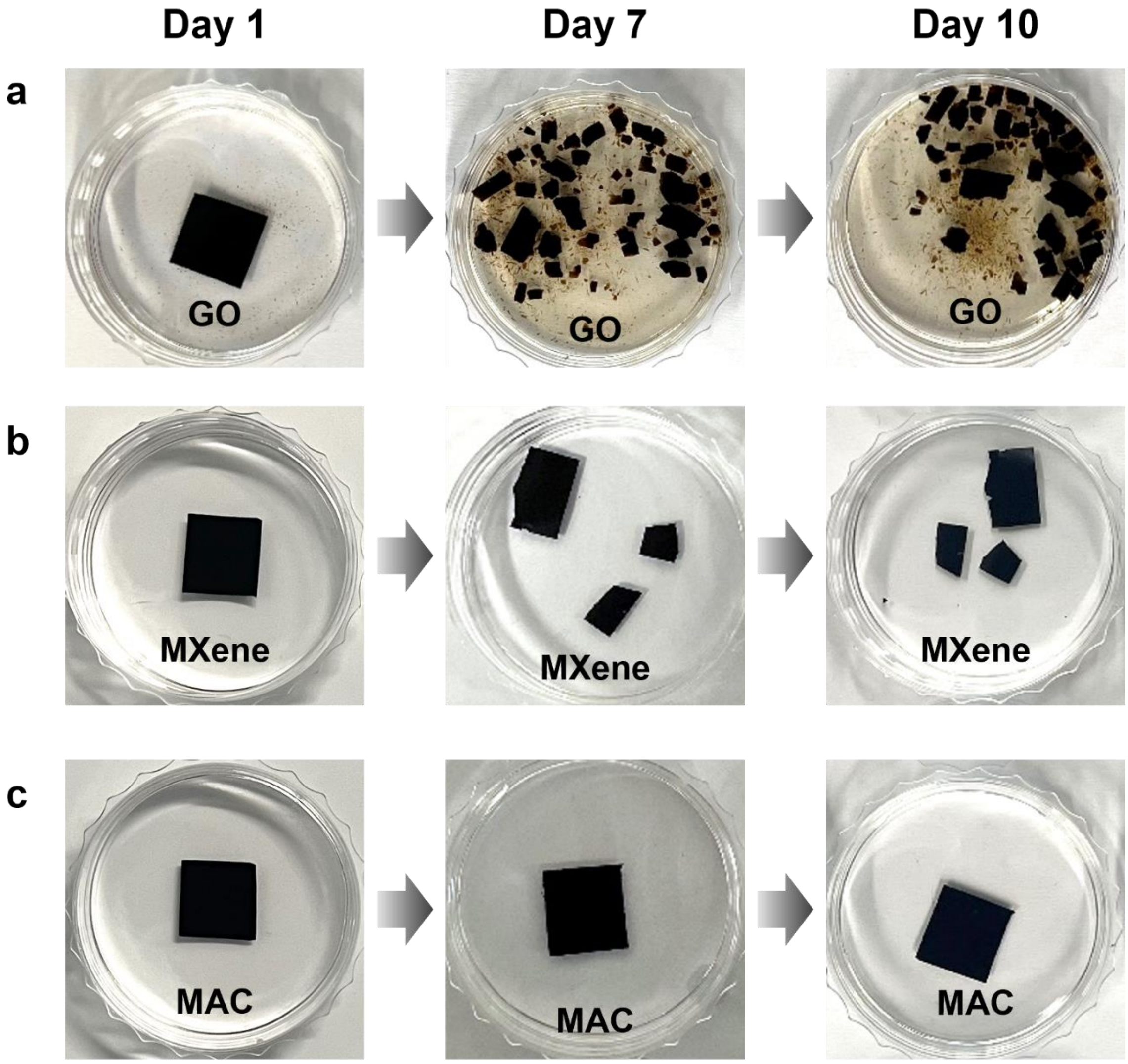

Supplementary Fig. 3. Aqueous stability of the membrane. Photographs of (a) GO, (b) MXene, and (c) the MAC membranes soaked in DI water for 1, 7, and 10 days. All the samples measure 1 × 1 cm^2.

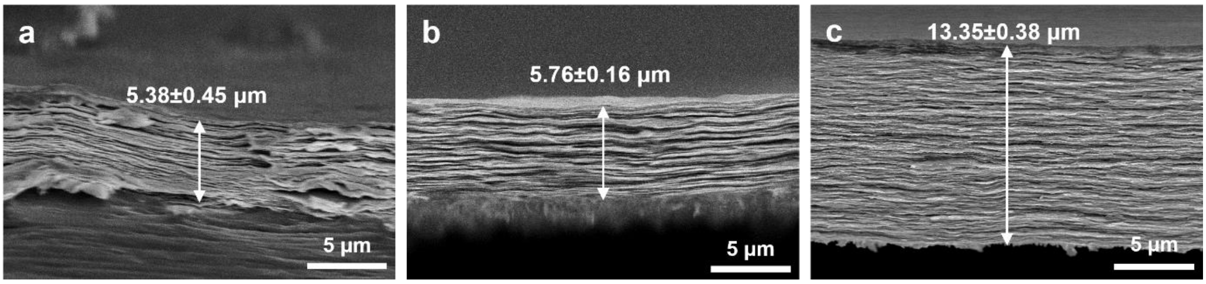

Supplementary Fig. 4. Cross-sectional SEM images of (a) MXene film, (b) MXene/AuNS, and (c) MAC membrane. All the films were prepared with the same amount of MXene (20 mg).

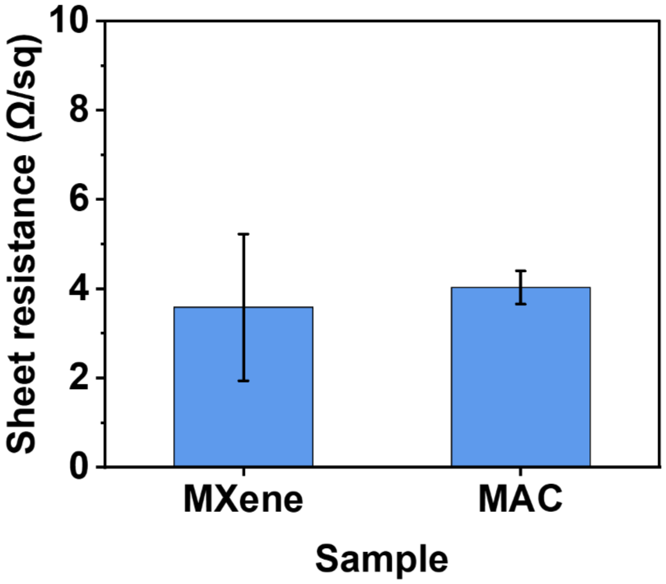

Supplementary Fig. 5. Sheet resistance of MXene and MAC film. The samples were prepared by MXene (20 mg) and MAC (7 wt% of AuNSs and 35 wt% of CNFs in 20 mg of MXene). Error bars represent standard deviations from three samples. Each sample was measured five times and the sizes are 1.5×1.5 cm^2. Source data are provided as a Source Data file.

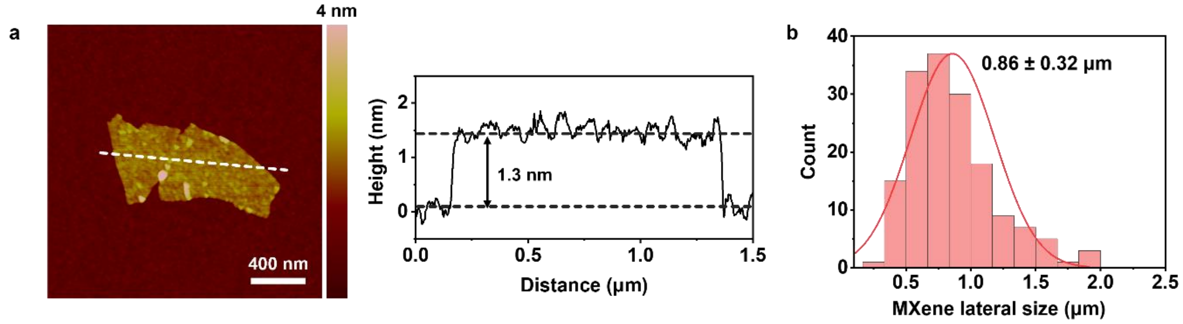

Supplementary Fig. 6. Characterization of the MXene nanosheets. (a) AFM image and cross-sectional plot of the MXene nanosheet. (b) Average lateral sizes of the MXene nanosheets. The histogram, mean, and standard deviation values in Supplementary Fig. 6b were obtained from 160 MXene nanosheets. Source data are provided as a Source Data file.

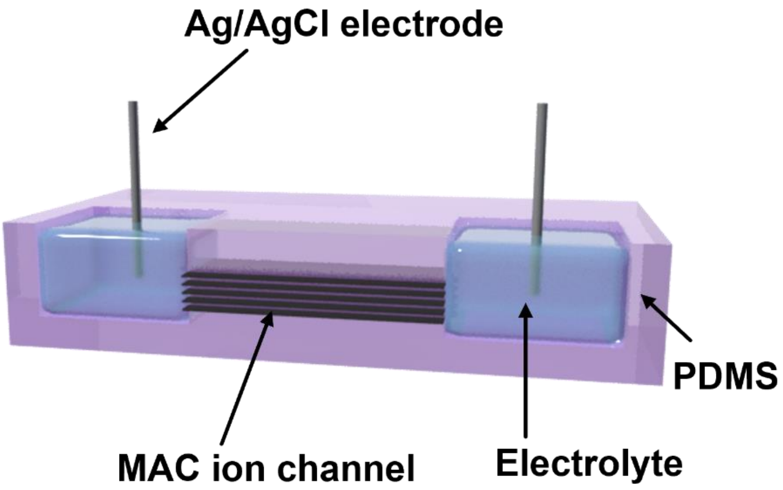

Supplementary Fig. 7. Schematic of the experimental setup for the observation of ion transport through the MAC membrane. The MAC membrane (0.5×1.5 cm^2) was encapsulated in PDMS elastomer, and each end was cut and opened to soak the MAC membrane with an equivalent concentration of KCl electrolyte (10^{-6} M to 1 M KCl).

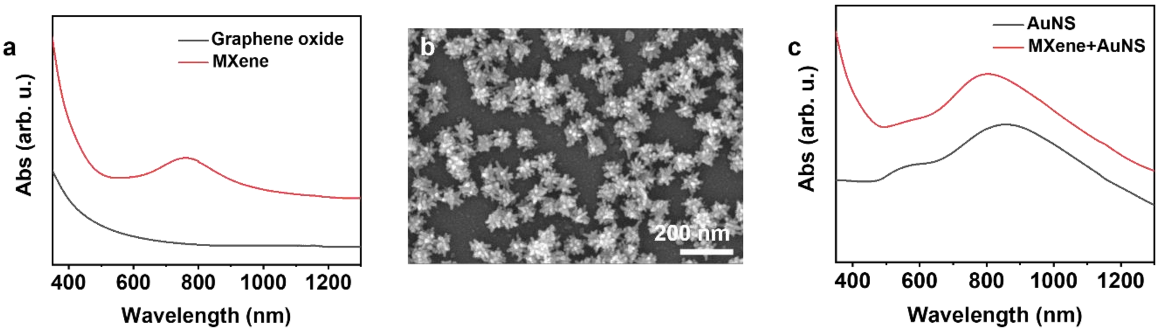

Supplementary Fig. 8. Photothermal properties of MXene and AuNS. (a) UV–vis–NIR absorbance spectra of graphene oxide and the MXene dispersion at the same concentration. (b) SEM image of AuNSs. (c) UV–vis–NIR absorbance spectra of AuNSs and MXene/AuNS dispersion. Source data are provided as a Source Data file.

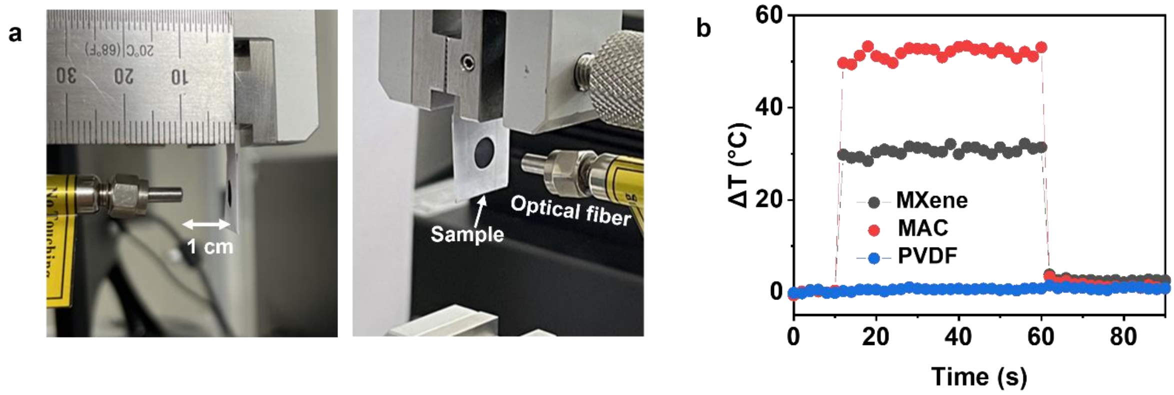

Supplementary Fig. 9. Photothermal effect of MXene and the MAC membrane. (a) Photographs of the experimental setup. (b) Temperature changes of MXene, MAC, and PVDF membrane under NIR laser irradiation at 84 mW cm^{-2}. ΔT represents the temperature difference between the sample and the surroundings. Each membrane measured 1 × 1 cm^2 and was thermally insulated in air to measure the temperature change. The distance between the optical fiber and the membrane was fixed at 1 cm. Source data are provided as a Source Data file.

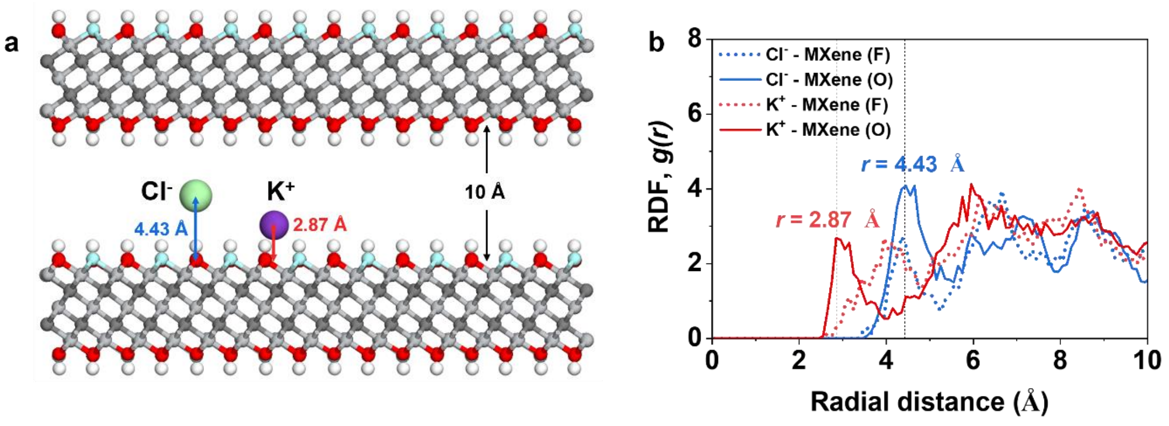

Supplementary Fig. 10. Ion distribution in the MXene nanochannel. (a) Schematic of ions in the MXene nanochannel. The K^+ and Cl^- ions are indicated by violet and green, respectively. (b) Radial distribution function (RDF) analysis between the ions and termination groups of MXene. Source data are provided as a Source Data file.

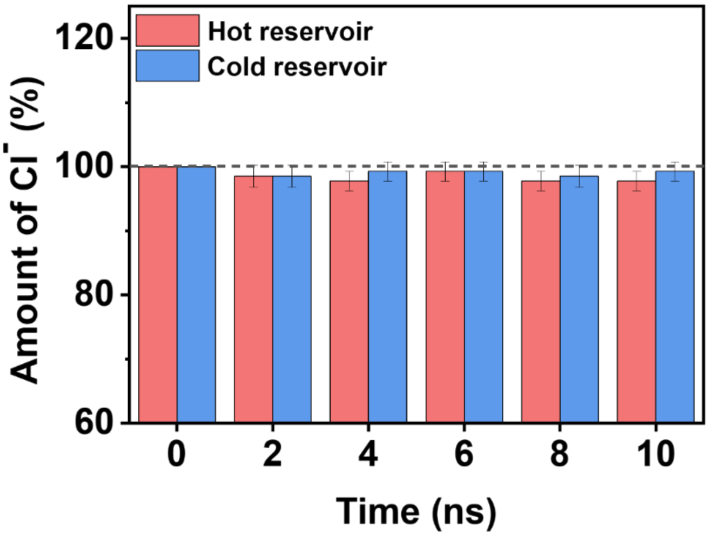

Supplementary Fig. 11. MD simulation of ion flow in the MXene nanochannel. Amount of Cl^- ions in each reservoir with time. Error bars denote standard deviation for 500 ps before each specified time. Source data are provided as a Source Data file.

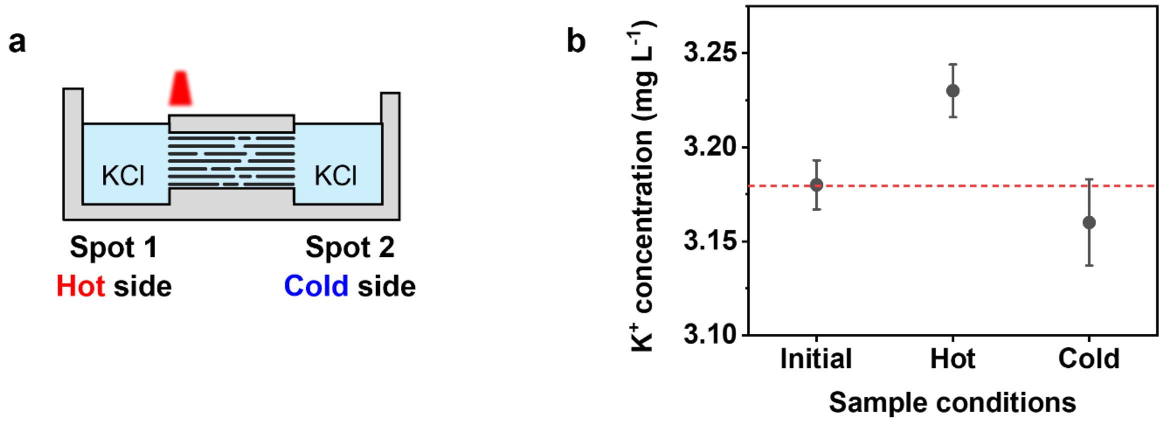

Supplementary Fig. 12. Inductively coupled plasma-optical emission spectrometry (ICP-OES) measurement. (a) Scheme of the experimental setup. (b) K^+ concentration of the initial solution, and the hot and cold sides measured using ICP-OES. Both electrolyte reservoirs contain 10^{-4} M KCl, and the MAC membrane near spot 1 is irradiated for 1 min (808 nm, 157 mW cm^{-2}). After light illumination, the electrolytes in spot 1 and spot 2 were collected for ICP-OES measurement. Error bars in Supplementary Fig. 12b represent standard deviation from three measurements (n=3). Source data are provided as a Source Data file.

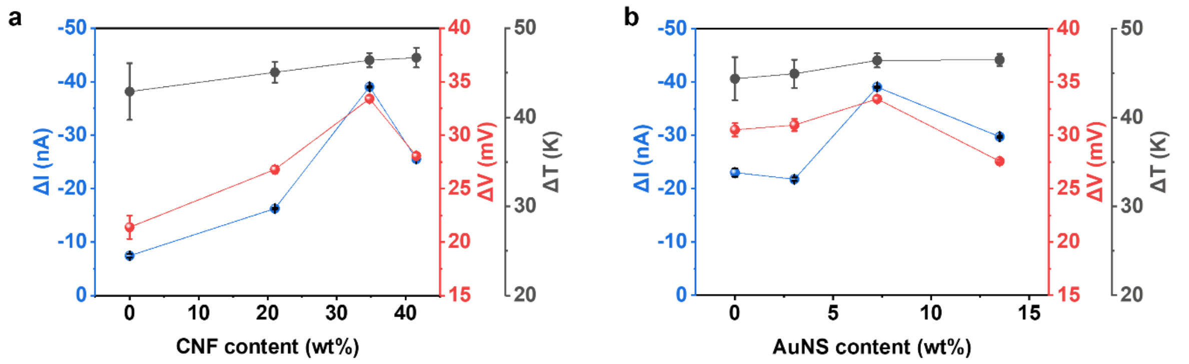

Supplementary Fig. 13. Photothermally induced current and voltage under (a) different CNF contents with fixed amounts of AuNSs (7 wt%) and (b) different contents of AuNSs with fixed amounts of CNFs (35 wt%). A fixed amount of MXene (20 mg) was used for all the sample conditions. The samples are soaked in 1 μM KCl electrolyte for the measurements. Error bars represent standard deviation from three samples, and all samples were exposed to the same intensity of NIR light (808 nm, 157 mW cm^{-2}). Source data are provided as a Source Data file.

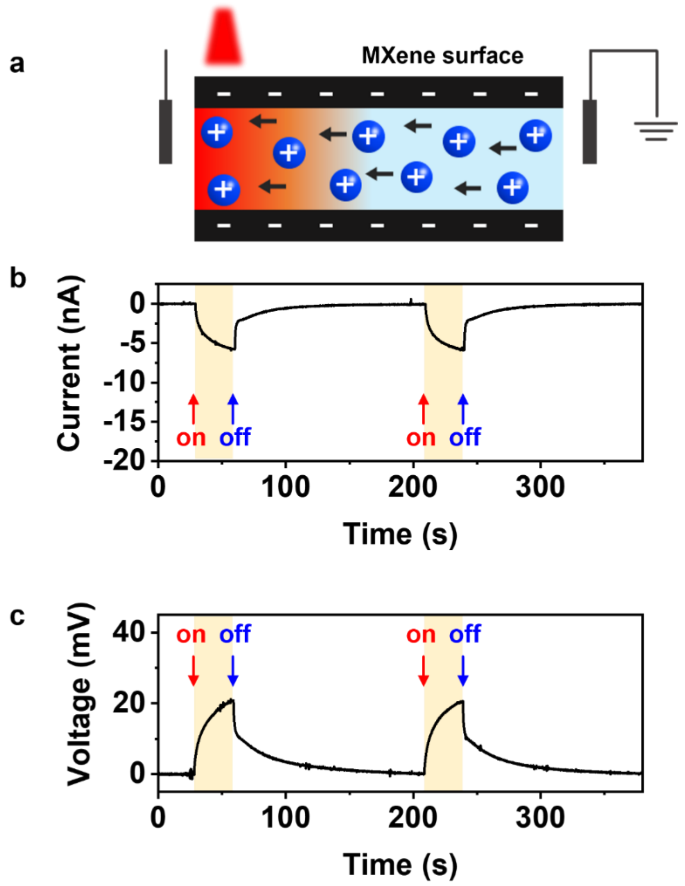

Supplementary Fig. 14. Photothermally induced current and voltage of the pristine MXene channel. (a) Scheme of the MXene ion channel under NIR light exposure. (b) Current and (c) voltage under NIR light on/off cycles (808 nm, 157 mW cm^{-2}). The MXene channels were soaked in 1 μM KCl electrolyte overnight for the measurements. Source data are provided as a Source Data file.

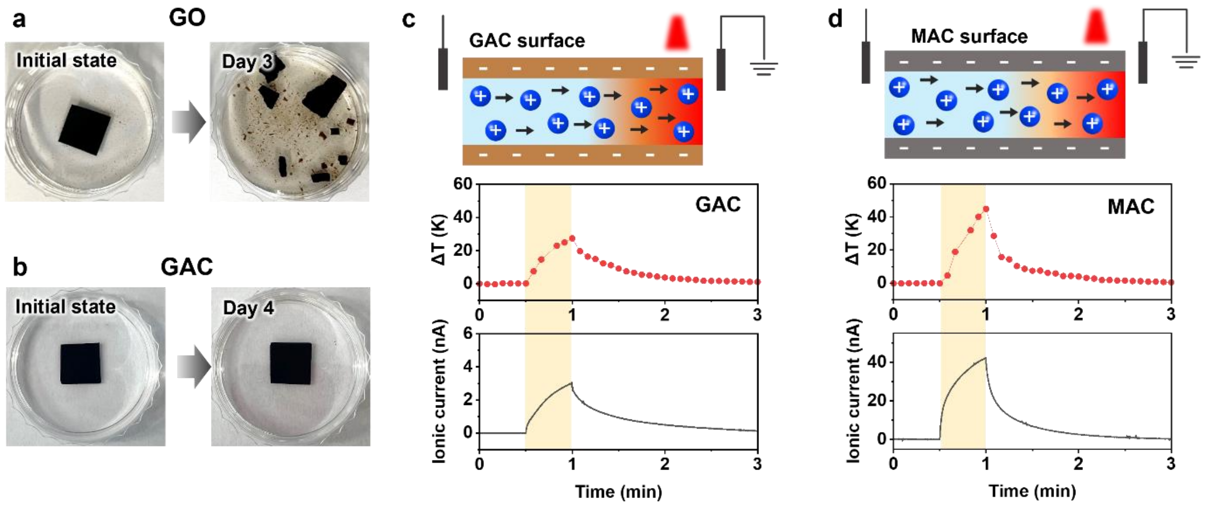

Supplementary Fig. 15. Aqueous stability and ionic current of GAC. Aqueous stability of (a) pristine GO and (b) GAC membrane. Ionic current of (c) GAC membrane and (d) MAC membrane under the same light intensity (157 mW cm^{-2}). GO and GAC were soaked in DI water (1×1 cm^2) for the aqueous stability test. The GAC and MAC membranes were soaked in 1 μM KCl electrolyte overnight for the ionic current measurements. Source data are provided as a Source Data file.

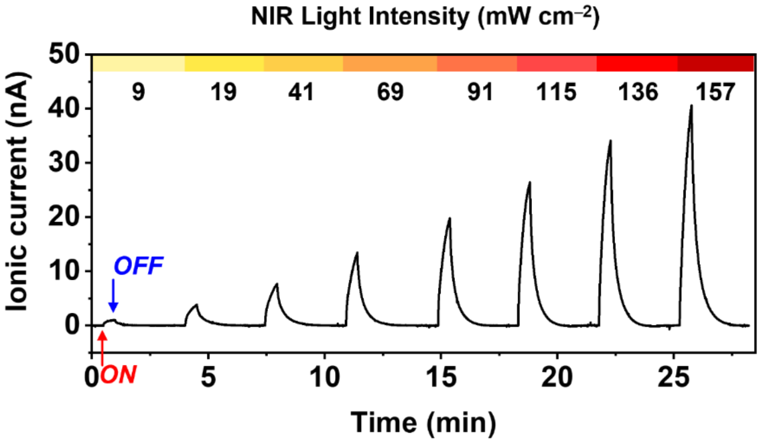

Supplementary Fig. 16. Photothermally induced ionic current of the MAC channel. Ionic current under increasing NIR light intensity. The MAC membrane was soaked in 1 μM KCl electrolyte overnight for the measurements. Source data are provided as a Source Data file.

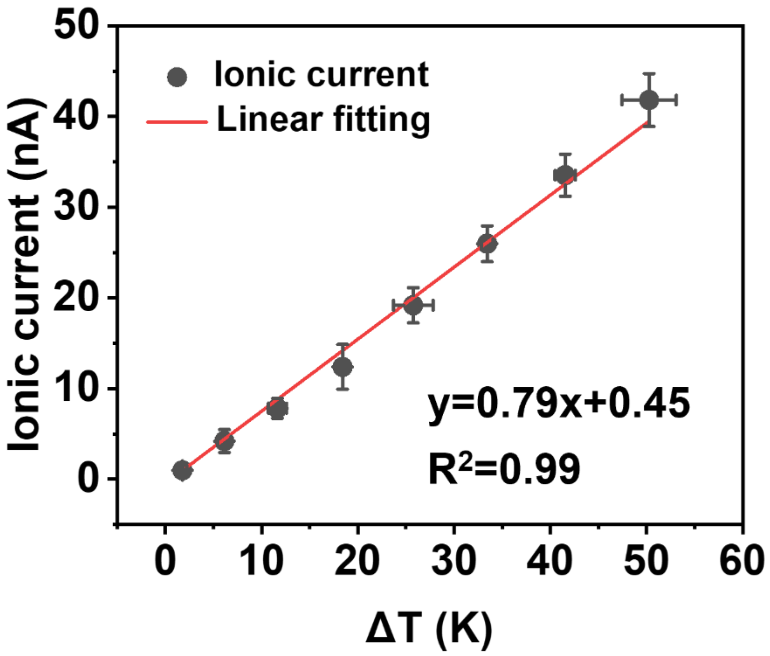

Supplementary Fig. 17. Photothermally induced ionic current of the MAC membrane under temperature changes. Error bars represent standard deviation from three samples. Source data are provided as a Source Data file.

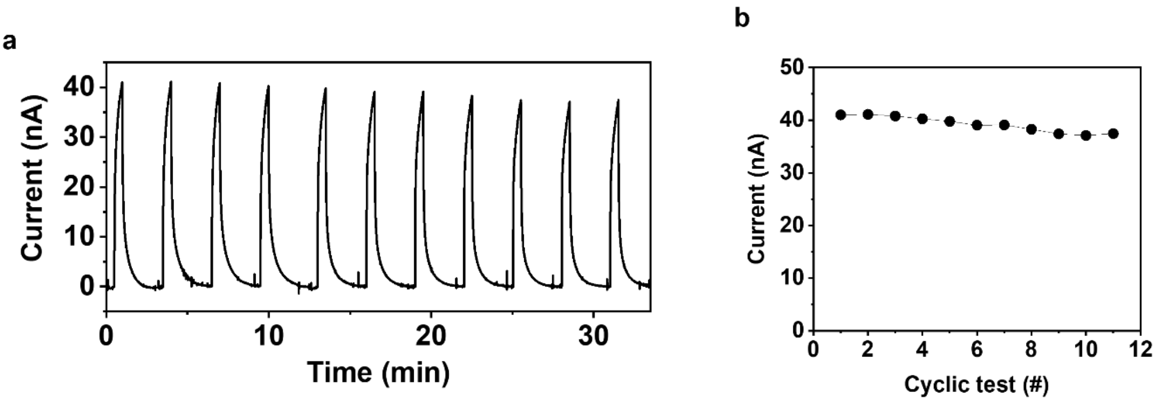

Supplementary Fig. 18. Cyclic ionic current of the MAC membrane. (a) Ionic current under the cyclic light on/off tests. Light (157 mW cm^{-2}) is irradiated for 30 s and switched off for the next 2.5 min for one cycle. (b) Ionic current after light irradiation for 30 s. The MAC membrane was soaked in 1 μM KCl electrolyte overnight for the measurements. Source data are provided as a Source Data file.

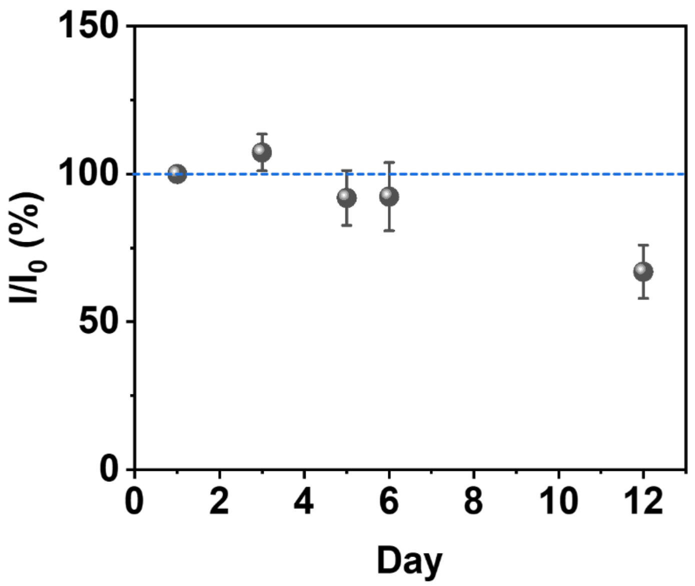

Supplementary Fig. 19. Long-term stability of MAC ion channel. Ionic current changes (I/I_0, %) of MAC membrane for 12 days soaked in 1 μM of KCl and stored in vacuum environment. I_0 is ionic current values obtained at day 1. Error bars represent standard deviations from 4 samples. Source data are provided as a Source Data file.

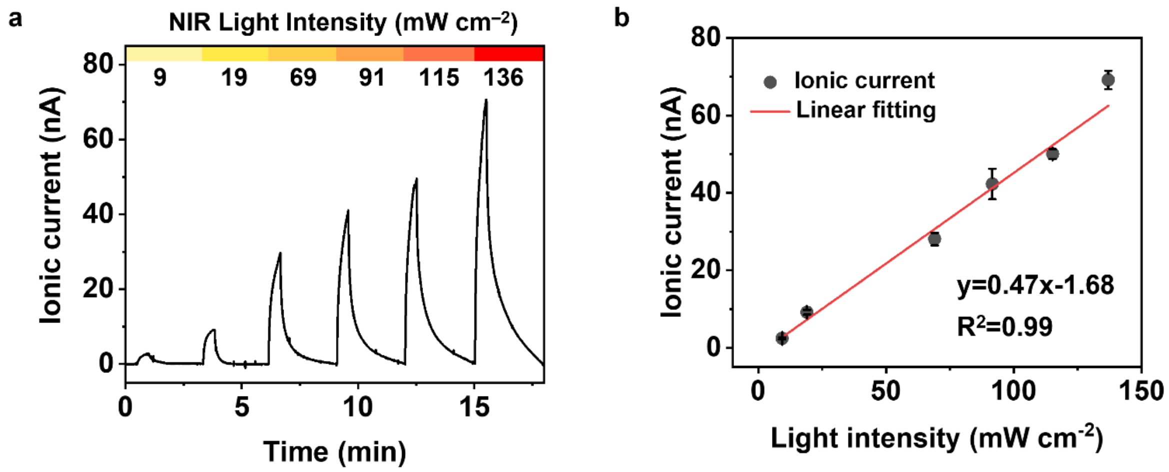

Supplementary Fig. 20. Photothermally induced ionic current of the ionogel–MAC channel. (a) Ionic current under increasing NIR light intensity. (b) Linearly fitted photothermal current of the ionogel–MAC channel under different light intensities. Error bars in Supplementary Fig. 20b represent standard deviation from three samples. The MAC membrane was soaked in 1 μM KCl electrolyte overnight and the pre-gel solution (acrylamide pre-gel solution with 1 μM KCl as the solvent) was polymerized as a solid electrolyte. Source data are provided as a Source Data file. Source data are provided as a Source Data file.

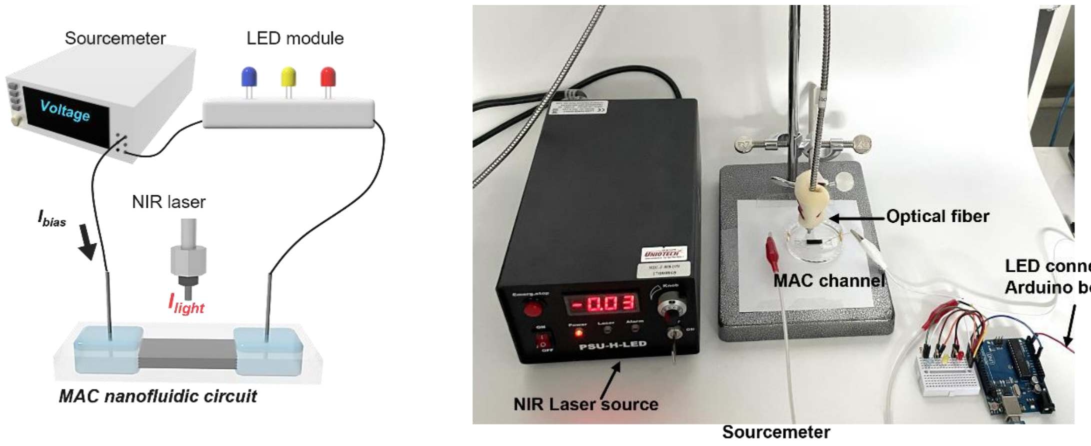

Supplementary Fig. 21. Experimental set-up for LED light switching. The sourcemeter, MAC channel, and LED module are connected to test the MAC nanofluidic circuit for switching light bulbs depending on two voltage thresholds (65 mV and 25 mV).

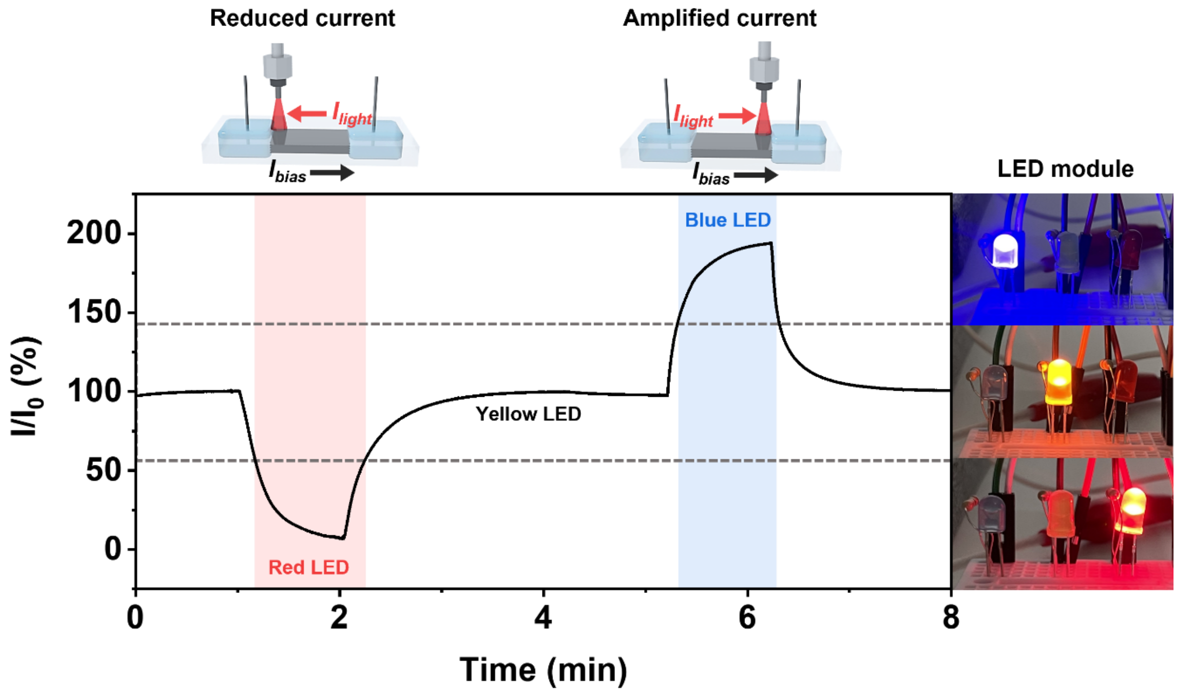

Supplementary Fig. 22. Ionic current under continuous voltage bias (45 mV). Reduced ionic current and amplified ionic current are monitored depending on the position of light irradiation. Source data are provided as a Source Data file.

Supplementary Table 1. Lennard–Jones parameters for the KCl solution and MXene channel. Note that the references indicate the force field used in this study.

Atoms	ε (kcal mol^{-1})	σ (Å)	q (e)
	MXene		
O of hydroxyl group on the surface[1]	0.1554	3.165541	−0.976
O on the surface[1]	0.1554	3.165541	−0.8235
H of hydroxyl group on surface[1]	0	0	0.497
Ti of MXene outer side[1]	0.6087	0.19565	1.490625
Ti of MXene inner side[1]	0.6087	0.19565	0.697188
C of MXene[1]	0.066	0.35	−1.20859
F on the surface[2]	0.1554	3.165541	−0.5508
	KCl solution		
O of water[3]	0.1554	3.165541	−0.82
H of water[3]	0	0	0.41
K ion[4]	0.1	3.33401	1.0
Cl ion[5]	0.1001	4.399971	−1.0
	Piston wall		
C[6]	0.070	3.39848	0

Supplementary Table 2. Recent studies on the photo(thermo)-responsive ionic current and voltage through a nanoconfined ion channel

Materials	Ionic current	Ionic voltage	Light source (range)
Graphene oxide (GO)[7]	2.27 nA (100 mW cm^{-2})	N/A	Xe lamp (20–100 mW cm^{-2})
Positively, negatively charged GO (p-GO, n-GO)[8]	1 nA (40 mW cm^{-2})	5.8 mV (40 mW cm^{-2})	Xe lamp (20–100 mW cm^{-2})
Partial bilayer GO[9]	6.4 nA (60 mW cm^{-2})	10.5 mV (60 mW cm^{-2})	Xe lamp (20–100 mW cm^{-2})
Janus GO[10]	14.5 nA (13.4 mW cm^{-2})	20.3 mV (13.4 mW cm^{-2})	UV lamp (λ=365 nm; 5–25 mW cm^{-2})
WS_2, MoS_2[11]	10.3 nA (94.1 mW cm^{-2})	31.1 (94.1 mW cm^{-2})	LED lamp (λ=470 nm; 30–94.1 mW cm^{-2})
Silica nanochannel, PET conical nanochannel[12]	N/A	0.71 mV K^{-1}	Thermal stimulus
$Ti_3C_2T_X$ MXene[13]	N/A	1 mV K^{-1}	Solar simulator (AM1.5G; 74–127 mW cm^{-2})
This work (MAC; MXene/AuNS/CNF)	40.8 nA (157 mW cm^{-2})	33.4 mV (157 mW cm^{-2})	NIR light (λ=808 nm; 9–157 mW cm^{-2})

Supplementary references

1 Kang, Y., Li, X., Tu, Y., Wang, Q. & Ågren, H. On the mechanism of protein adsorption onto hydroxylated and nonhydroxylated TiO2 surfaces. *The Journal of Physical Chemistry C* **114**, 14496-14502 (2010).

2 Muckley, E. S. *et al.* Multimodality of structural, electrical, and gravimetric responses of intercalated MXenes to water. *ACS nano* **11**, 11118-11126 (2017).

3 Cygan, R. T., Liang, J.-J. & Kalinichev, A. G. Molecular models of hydroxide, oxyhydroxide, and clay phases and the development of a general force field. *The Journal of Physical Chemistry B* **108**, 1255-1266 (2004).

4 Koneshan, S., Rasaiah, J. C., Lynden-Bell, R. & Lee, S. Solvent structure, dynamics, and ion mobility in aqueous solutions at 25 C. *The Journal of Physical Chemistry B* **102**, 4193-4204 (1998).

5 Smith, D. E. & Dang, L. X. Computer simulations of NaCl association in polarizable water. *The Journal of Chemical Physics* **100**, 3757-3766 (1994).

6 Wang, L., Dumont, R. S. & Dickson, J. M. Nonequilibrium molecular dynamics simulation of pressure-driven water transport through modified CNT membranes. *The Journal of Chemical Physics* **138**, 124701 (2013).

7 Yang, J. *et al.* Photo-induced ultrafast active ion transport through graphene oxide membranes. *Nature communications* **10**, 1-7 (2019).

8 Quan, D. *et al.* Laterally Heterogeneous 2D Layered Materials as an Artificial Light-Harvesting Proton Pump. *Advanced Functional Materials* **30**, 2001549 (2020).

9 Zhang, Y. *et al.* Photoinduced Directional Proton Transport through Printed Asymmetric Graphene Oxide Superstructures: A New Driving Mechanism under Full-Area Light Illumination. *Advanced Functional Materials* **30**, 1907549 (2020).

10 Wang, L. *et al.* Light-Driven Active Proton Transport through Photoacid-and Photobase-Doped Janus Graphene Oxide Membranes. *Advanced Materials* **31**, 1903029 (2019).

11 Jia, P. *et al.* Harnessing Ionic Power from Equilibrium Electrolyte Solution via Photoinduced Active Ion Transport through van-der-Waals-Like Heterostructures. *Advanced Materials* **33**, 2007529 (2021).

12 Chen, K., Yao, L. & Su, B. Bionic thermoelectric response with nanochannels. *Journal of the American Chemical Society* **141**, 8608-8615 (2019).

13 Hong, S. *et al.* Photothermoelectric Response of Ti3C2T x MXene Confined Ion Channels. *ACS nano* **14**, 9042-9049 (2020).
