# Supplementary material for: Photosensitive ion channels in layered MXene membranes modified with plasmonic gold nanostars and cellulose nanofibers
Source: Nat Commun. 2023 Jan 23;14:359. doi: 10.1038/s41467-023-36039-5 (PMC9870870; doi:10.1038/s41467-023-36039-5)
Supplement: Supplementary file 2 — Description of Additional Supplementary Files [file 41467_2023_36039_MOESM2_ESM.pdf]

Description of Additional Supplementary Files

File Name: Supplementary Movie 1

Description: LED light shifting from yellow to red under NIR light (opposite direction of Ilight to Ibias).

File Name: Supplementary Movie 2

Description: LED light shifting from yellow to blue under NIR light (same direction of Ilight to Ibias).
